# Insect Decline—Evaluation of Potential Drivers of a Complex Phenomenon

**DOI:** 10.3390/insects15121021

**Published:** 2024-12-23

**Authors:** Michael E. Grevé, Michael Thomas Marx, Sascha Eilmus, Matthias Ernst, John D. Herrmann, Christian Ulrich Baden, Christian Maus

**Affiliations:** Bayer AG, 40789 Monheim, Germany

**Keywords:** agricultural intensification, pesticides, landscape management, urbanization, renewable energy, bioenergy crop, permanent grassland

## Abstract

The decline of insects is a global concern, yet identifying the factors behind it remains challenging due to the complexity of potential drivers and underlying processes, and the lack of quantitative historical data on insect populations. This study assesses 92 potential drivers of insect decline in West Germany, where significant declines have been observed. Using data from federal statistical offices and market surveys, the study traces changes in landscape structure and agricultural practices over 33 years. Over the years, the region underwent major landscape changes, including reduced cropland and grassland and increased urbanization and forest areas. Potential detected drivers of insect decline include: (1) urban expansion, reducing insect habitats as urban areas increased by 25%; (2) intensified grassland management; (3) shifts in arable land use towards bioenergy and feed crop cultivation, particularly corn, driven by dairy farming intensification and renewable energy policies. While the toxic load of pesticide application has decreased, land-use changes, most likely driven by market demands and shifts in national and EU policies, have reduced habitat availability and suitability for insects. This study highlights how these landscape and land management changes over the past 33 years align with the observed decline in insect biomass in the region.

## Simple Summary

Insect decline is a global environmental issue. Its causes are not yet fully understood due to the complexity of contributing factors, and a lack of historical data. Here, we investigate a set of 92 potential drivers and their development over the past decades, in connection with a dataset on insect biomass decline in Western Germany over 33 years. Among potential drivers, factors related to landscape structure and land management changes were most significant. Besides increasing urbanization, grassland management intensification, shifts in arable land use towards bioenergy and feed crop cultivation, and dairy farming intensification are among the likely key factors linked to insect decline in the region. Conversely, no obvious links were found to climate and weather, or to crop protection. Our results suggest that land management intensification can be considered an important driver of insect decline in central Europe, rather than land-use change, which is largely restricted to urbanization. Consequently, improved landscape management, including an optimized balance between extensively and intensively managed areas and natural habitats, and conservation of structural landscape diversity could offer solutions to counteract insect decline. Our results will help facilitate more targeted and focused action against insect decline by highlighting likely causative factors.

## 1. Introduction

Insect decline is a complex phenomenon that has currently been reported globally and entails declines in a variety of dimensions, such as insect abundance, biomass, and biodiversity [1,2,3]; however, the decline is not consistent across regions, habitats, and taxa [4,5]. In the past years, it has been a topic of intensive research and was reviewed and highlighted in studies like Cardoso et al. [6] and Wagner et al. [7]. Wagner et al. [7] particularly emphasize the multifactorial nature and complexity of possible causes of insect decline.

The decline of insects is best documented in central, western, and northern Europe across different insect groups and different habitats [8,9,10,11,12,13]. A publication which gained strong attention in the scientific community, and which raised public and political awareness regarding the issue, is Hallmann et al. [14]. The authors showed a biomass reduction of 75% and more in flying insects in German nature conservation areas over nearly three decades.

Understanding the drivers of insect decline is a complex endeavor, as it involves disentangling the interplay of numerous factors and the varied responses of indicators like species richness, biomass, and abundance of environmental stressors [15]. Short-term studies and snapshot surveys can provide useful insights but may not fully capture the broader trends [16,17,18,19], given the natural fluctuations in insect populations over time [20,21]. A key challenge in analyzing insect decline is the scarcity of long-term data. To study population or community trends effectively, extended data series from consistent locations are essential to understand changes and their environmental context [22]. This can be achieved by generating new monitoring data or evaluating existing datasets; however, while new data collection allows direct measurement of relevant environmental factors, it requires significant time to yield meaningful results. Systematic insect monitoring efforts in Europe over recent decades remain limited (e.g., van Swaay et al. [23]), and newer initiatives [24,25,26,27] are still in early stages, making them unsuitable for analyzing past declines.

Utilizing historic data can provide valuable insights into past trends [22] but has its own challenges, as their availability is limited due to the typically relatively short duration of academic projects and challenges with historical data consistency. Private initiatives can be a valuable complement to public monitoring projects (e.g., Fontaine et al. [28]), but they frequently focus on qualitative rather than quantitative aspects. Addressing the paucity of baseline data remains a critical challenge in studying insect decline, hindering our understanding of past insect population abundance and diversity trends. Montgomery et al. [19] also emphasized this issue, highlighting the need for more comprehensive data to enhance our knowledge in this area. However, the few relevant datasets existing [5] are of great value for the ex-posteriori analysis of decline processes.

Among the driving factors that have been implicated in insect decline, habitat availability, condition, and fragmentation seem to play a particularly important role. Habel et al. [17], for example, reviewed studies with a European focus that showed either reduced species richness, losses of biomass, or insect abundances and concluded that agricultural intensification is leading to habitat loss and reduced functional connectivity. Many of the environmental factors which may act as stressors causing insect decline have already been widely studied, such as land-use and grassland management intensification [29,30,31,32]. Other factors, like increasing biofuel crop production or wind turbines, have been found to negatively affect local biodiversity [33,34] but are less considered. Further potential factors, such as use of pesticides [35], are commonly assumed to be relevant stressors but have rarely been systematically investigated due to a lack of detailed historic data. Other stressors that act over longer time periods require long-term monitoring efforts, such as urbanization (multiple years) [36,37] or climate change (multiple decades), [38,39] which makes them challenging to investigate. Consequently, most studies are either individual case studies or focused on single factors [40], and the outcome of studies with single explanatory factors should be interpreted cautiously [41].

In this study, we analyze various environmental factors that might drive insect decline in a region in Western Germany (North Rhine-Westphalia, NRW). Using long-term data on flying insect biomass (FIB) collected in conservation areas (of varying size and protection status, with over 50% of the locations being sampled only once) from Hallmann et al. [14], collected at 89 individual sampling events at 59 locations between 1989 and 2016 and two locations from Mühlethaler et al. [42] from 2020 and 2021, we investigate potential correlations between regional insect decline and various environmental factors.

Despite potential challenges with the dataset’s consistency and comparability [43], we consider it safe to assume that the detected decline can be seen as representative of the decline of flying insect biomass in western Germany, as elucidated by Hallmann et al. [14]:

“Although the present dataset spans a relatively large number of years (27) and sites (63), the number of repetitions (i.e., multiple years of seasonal distributions at the same locations) was lower (*n* = 26). We are however confident that our estimated rate of de-cline in total biomass resembles the true rate of decline, and is not an artifact of site selection. […]. Secondly, using only data from sites at which malaise traps were operating in at least two years, we estimated a rate of decline similar to using the full dataset […], with the pattern of decline being congruent across locations […]. Taken together, there does not seem to be evidence that spatial variation (between sites) in this dataset forms a confounding factor to the estimated temporal trend, and conclude that our estimated biomass decline is representative for lowland protected areas in west Germany”.

Hallmann et al. [14] could not directly link the detected decline in flying insect biomass to specific factors like land use, habitat type, or habitat characteristics in the protected areas. Instead, they suggest that more wide-ranging factors were likely involved. Hence, our study focuses on land use changes, habitat characteristics, and other potential stressors on a larger scale. Obviously, an optimal approach for this would be an in-depth analysis of changes in a rather narrow radius around individual sampling sites. However, for the Hallmann et al. [14] dataset, this was technically not feasible: Information on many of the parameters analyzed is only (publicly) available at a larger scale (e.g., the federal state level), and though some aspects of broader land-use change can nowadays be assessed with a fair amount of certainty using satellite imagery (e.g., Holmes et al., [44]), there are major challenges to applying this approach retrospectively back to the 1980s and 90s. Information on how various aspects of land use changed on a smaller scale up to three decades ago does not exist to our knowledge. However, since the small conservation areas studied by Hallmann et al. [14] are distributed and embedded in cultivated landscapes all over the federal state of NRW [45], we consider an appropriate hypothesis to be that factors determined at a state level will likewise affect the local conditions of a representative majority of the sampling sites.

Making use of the biomass data of Hallmann et al. [14] as a starting point, the objectives were to compile long-term data on 92 land use, agricultural practice, animal husbandry, pesticide use, and climatic change factors for the time period of the Hallmann et al. [14] study in NRW, and to systematically evaluate these factors on a regional level, linking them to quantifiable insect decline data from the same area on a per-year basis.

To achieve this, we leverage extensive datasets from publicly available databases, as well as farmer questionnaires on pesticide use covering the timespan of Hallmann et al. [14] and Mühlethaler et al. [42]. By doing so, we hope to contribute to a better understanding of the key factors responsible for the significant insect decline in the region.

## 2. Materials and Methods

For this study, time series data of various variables on factors that have been suggested as potential drivers behind insect decline and which are detailed in the following were gathered or acquired from multiple sources. The geographical scale of the data is the federal state of North Rhine-Westphalia (NRW) in Western Germany which has an area of roughly 34,000 km^2^ and is about the size of the neighboring countries Belgium (30,500 km^2^) or the Netherlands (41,500 km^2^). Where available, data from 1989 to 2022, which cover and exceed the study period of Hallmann et al. [14] and Mühlethaler et al. [42], were used. The prerequisites for selection and use of the data were high traceability and standardized data generation, as described below.

### 2.1. Land Use and Farming Data

All information in this section is summarized data per year for the whole of NRW as provided by the different public sector entities listed below.

Information on land coverage by urban areas and forests was provided by the German Land Registry Administration [46]. Information was published irregularly until the year 2007 and published annually after 2008 [47].

Data on land use and management, which included the areas used for the farming of different crop types and different grassland types, were obtained from annually published reports by the Federal Statistical Office of Germany. The main survey of land use by agricultural operations in Germany is conducted annually, making recourse of 80,000 individual surveys across the country. In addition, the following datasets were extracted from the Federal Statistical Office of Germany [47] for the dataset used in our study: the covers of permanent grassland (meadows, pastures and mowing pastures, and extensive grassland), fallow land, and relevant crops grown in NRW, the total yield, and yield per hectare of the largest crops. Additionally, the number of agricultural operations (farms) cultivating crops and permanent grassland in NRW, and information on the total stock of cattle, dairy cows, pigs, and sheep, and on animal products were included. While most variables were available on an annual basis, some information, such as the number of barns with dairy cows, is published only every other year. As information on the number of dairy cows and total tons of milk produced was not available for the years 2016 to 2021, this information was taken from the homepage of the association of dairy farming in NRW [48]. From a second annually published report by the Federal Statistical Office of Germany [49], information on nitrogen and phosphate fertilizer use were extracted that are based on domestic sales.

A detailed list of all variables and sources can be found in Supplementary File S1. The data provided by the Federal Statistical Office of Germany was subject to some variation in how variables were grouped. Details of which variables were combined to maintain consistency over the 33 years are stated in Supplementary File S1 as well.

### 2.2. Renewable Energy Data

Information on renewable energy generation for every year is provided on request by the Federal State Environmental Ministry of North Rhine-Westphalia. The State of NRW received the information from the local Transmission System Operator “Amprion”. The data used in this study covers bioenergy plants and onshore wind turbines, with the years of commissioning and the generated power known for each plant and turbine (data summarized and included in Supplementary File S1). Wind turbines which were subsequently taken out of service were included in the dataset for the years of service only.

### 2.3. Pesticide Data

There is no systematic, detailed data available on the use patterns of pesticides in Germany [50]. Pesticide sales data have been regularly published by the Federal Office of Consumer Protection and Food Safety (BVL) since 1987 (BVL, 2023), however, their limitation in the context of our data analysis approach is that a pesticide is not necessarily used in the year and in the place where it has been sold. The same is true for sales data available at pesticide manufacturers, which, moreover, are even more restricted to the products of their respective portfolios. To avoid the analysis being compromised by these uncertainties, we gathered datasets (described below) which refer to the application of pesticides rather than to sales, as the former is considered a more accurate proxy for insect exposure than the latter.

#### 2.3.1. Foliar (Spray) Uses of Pesticides

For information on pesticide applications in NRW, long-term market survey data on pesticide use by farmers was purchased. The data was generated by Kynetec, (Lüdinghausen, Germany, formerly Kleffmann Group), a market research institute, and is based on questionnaires completed by local farmers. The data includes information on which pesticide product was applied to which crop type with the respective tonnage and treated area for each crop type. It further includes information on the total area in which a farmer has been cultivating the specific crop for each year.

The data were available from 1995 onwards, with the first comprehensive dataset generated in 1996. As the 1995 dataset was still rather incomplete, we used the data from 1996 onwards.

Due to the nature of the surveys, the available data do not completely cover all pesticide applications in NRW, but they were used to draw a representative picture of how pesticide use changed over time. Similar data from the same market research institute was also recently used in a similar study by Van Deynze et al. [51].

The insecticide, fungicide, and herbicide products combined accounted for at least the 95th percentile of all data entries, and respective treated areas were included in the evaluation. This corresponded to 75 (out of a total of 151) insecticide products, 224 (out of a total of 465) fungicide products, and 407 (out of a total of 916) herbicide and plant growth regulator products. For each analyzed product, we extracted the active substances and relative contents (e.g., gram active substance per liter of product) in the products from the databases of pesticide distributors or their producers. The products that could not be assessed were predominantly single entries, often referring to old products which were withdrawn from the market years ago, and for which no valid information on the active substances was available.

The data on pesticides are shown in an aggregated form, i.e., not broken down to the level of the individual data point, to comply with the terms and conditions of use as stipulated by the supplier. However, this did not affect the data analysis since the aggregated data still provided the necessary granularity.

We employed this approach to assess the overall toxic load posed by the applied pesticides in total to non-target arthropods to conduct the data analysis. We consider this approach more meaningful than taking the individual per-product measurements because it enables a holistic view of pesticide toxicity through the evaluation of pesticide data in an aggregated form, taking into account the intrinsic toxicity of the evaluated products to non-target arthropods, as well as the treated area and the number of applications per crop as a proxy for arthropod exposure.

The overall toxic load of the applied pesticides was determined as follows: To obtain standardized ecotoxicological endpoints on the intrinsic toxicity of the individual products, we used LR50 values (in g active substance/ha) from standard laboratory tests with non-target arthropods *Aphidius rhopalosiphi*, a parasitoid wasp, [52] and *Typhlodromus pyri*, a predatory mite [53]. Both species are representative, highly sensitive standard testing organisms which are routinely tested as surrogate organisms in the initial steps of the risk assessment for most pesticide products according to EU regulations [54]. For our analysis, we used the lower (and thus more conservative) endpoint of both organisms for the respective active substance.

The laboratory tests are a worst-case testing system in which the organisms are exposed to pesticides that are freshly applied on glass plates and serve to determine the intrinsic toxicity potential of the test substance in a highly standardized way. In spite of the unrealistic design of the glass plate studies, we based our calculations on their results for three reasons: first, to go with the most conservative approach, second, the endpoints of these studies are available for most pesticides, and third, the aim of the exercise was not to determine potential risks under realistic exposure conditions, but to do a comparative calculation of relative toxic loads to which non-target arthropods have been exposed over the years.

The endpoints are publicly available in the pesticide properties database (PPDB) of the University of Hertfordshire [55]. The PPDB collects and stores endpoints of EU regulatory and evaluation data by the European Commission, European Food Safety Authority (EFSA), and European Medicines Agency. In cases where endpoints were not available in the PPDB, the regulatory endpoints were taken from the EFSA conclusions for the respective active substance.

In cases when no glass plate studies were available, the lowest endpoints of the next higher tier of testing (e.g., extended laboratory tests with exposure of the test organism exposed to the pesticide on a natural matrix, like leaf surfaces) with a more realistic exposure design were used. The direct comparison of the endpoints of different study tiers (e.g., laboratory vs. extended laboratory) may, strictly seen, be considered debatable, since higher tier studies tend to have higher endpoints, compared to the corresponding lower tier approaches. We nevertheless chose this approach given that the impact on the data analysis and assessment is lower than excluding data from the analysis, which would reduce significantly the robustness of the analysis.

A limited number of active substances which were on the market before the current regulatory testing system was established, and were not registered again later on, could not be included in the toxic load analysis. Hence, the toxic load of pesticides in the early years of the analysis may be underestimated to some degree. The list of endpoints and their source are available in Supplementary File S2.

With the datasets of the market survey, the ecotoxicological endpoints for the non-target arthropod species, and the cultivated area of the respective crops in NRW, we calculated the total amount of pesticide (tons per hectare) used per crop type and year in NRW, as well as the resulting toxic load for arthropods of each active substance (unitless) weighted by the proportion of treated crop area in NRW per crop and year. A detailed description of the analyses including an example is available in Supplementary File S3. In the analyzed period, 1434 products with more than 200 active ingredients were applied in the studied region.

#### 2.3.2. Seed Treatment Uses of Pesticides

Use data for seed treatment applications are particularly challenging to obtain. Like foliar uses, no information on seed treatment product use is publicly available. Moreover, seed treatment use data was not assessed by the Kynetec market research. And finally, use data for seed treatment products are by far more difficult to assess than for foliar treatments: on the one hand, seed treatment may be brought into the field at completely different times and places from those where the products were sold, and where the seeds were treated. And on the other hand, the farmer is not directly purchasing and applying a specific seed treatment product, but usually acquires and plants the pre-treated seeds with a given composition of active substances. Therefore, seed treatment use can, even as an approximation, hardly be derived from sales data.

Given that seed treatment is an agricultural practice that has been used during the assessed time in the studied region, we considered it would not be accurate to exclude any type of information about these pesticides uses in our assessment. However, as the data for pesticides used for seed treatment are not as accurate as the ones for foliar treatments, we had to rely on estimates for seed treatment usage.

For the analysis, to the best of our knowledge, all insecticidal seed treatments that were approved by the Federal Office of Consumer Protection and Food Safety (BVL) in Germany for rapeseed, cereals, sugar beets, corn, and potatoes (which are the main crops that receive seed treatment) since 1995 were recorded. Whether and which seed coatings were approved before 1995 could be reconstructed with an appropriate level of certainty.

In total, 46 different seed treatment products have been approved since 1995. For each product, the approval number, start date of approval, end date of approval, and expiration date were determined (Supplementary File S4). The expiration date marks the date after which an application of a product is no longer permitted in Germany. Additionally, the active ingredients content of a product, maximum registered application rate per active ingredient and hectare, and crop were identified. The amounts applied were calculated according to the assumptions outlined below, starting on the date of approval until the expiration date. In the few cases where products were approved after 1 October, the calculation for the products was done starting with the following year, as the planting of seeds treated with the product after this date is not likely.

Since there are no sales figures available for the different seed treatment products, the actual shares of a product of the total applied area could not be calculated. As a most conservative approximation, we assumed the application of seed treatments on the entire cultivation area of a crop and calculated the treated cultivated area for each product proportionally. If only one product is approved for a crop in a given year, we assumed the application of that product to the complete area cultivated with the crop. If five products were approved in a year for the same crop, each product was assumed to be applied to 20% of the total cultivated area.

Since foliage-dwelling species are not normally exposed to seed treatment products, in contrast to soil-dwelling non-target arthropods, tests on the forage-dwelling species listed above do not belong to the regulatory requirements for seed treatment products in the EU according to the SANCO/10553/2012 draft document [56]. Since, accordingly, lower-tier endpoints of *A. rhopalosiphi* and *T. pyri* were not readily available for many seed treatment products, endpoints for the soil organisms *Folsomia candida* [57], a collembolan species, and *Hypoaspis aculeifer* [58], a soil mite, were used to calculate the toxic loads of the respective products. Here, the respectively lower NOEC (no observed effect concentration) value for both species was used for the calculation. The list of endpoints and their source are available in Supplementary File S2. For the calculation, the total estimated tonnage of each active substance was divided by the regulatory endpoint to determine the toxic load index for seed treatments.

Since there are no herbicidal seed treatments, and fungicidal products can be assumed to have a negligible influence on the overall toxic load, as could be seen in the evaluation of the foliar products, we focus here on insecticidal seed treatment products. As the number of chemistries used in seed treatment products, compared to foliar products, is limited, we depict the toxic load of seed treatments broken down into their chemical class.

### 2.4. Weather Data

For weather data, we extracted monthly total precipitation and average air temperature information from the Climate Data Center (CDC) [59] provided by Germany’s National Meteorological Service (Deutscher Wetterdienst—DWD) for NRW for the years 1989 to 2022.

Recent studies showed that insects appear to be especially sensitive to precipitation and temperature in winter months [41], but weather in all seasons can affect insects [60]. Hence, for the selection of weather parameters analyzed, we focused on the mean temperature and the total precipitation separately for all four seasons with winter from December to February preceding the samplings season, spring from March to May, summer from June to August and fall from September to November. This variable selection does not cover local extreme weather events, but longer events like droughts were covered.

### 2.5. Normalization of the Biomass Dataset

**Figure 1:**
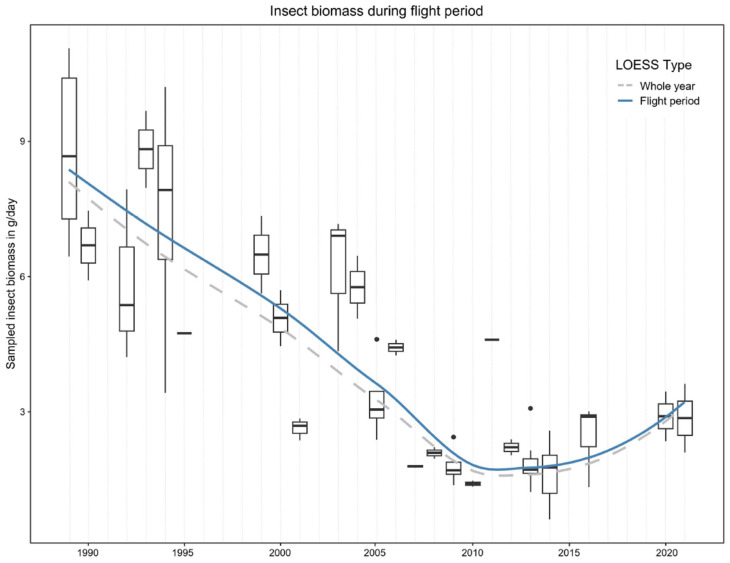
Mean (sampled) biomass of flying insects (in grams per day, FIB) during the insect flight period (April to September) is presented based on the biomass data derived from Hallmann et al. [14] and Mühlethaler et al. [42]. Each data point represents the mean insect biomass sampled per location. The depicted graph illustrates the LOESS correlation curves for the specific insect flight period and the entire sampling duration as reported in Hallmann et al. [14] study. The data for the years 2020 and 2021 from Mühlethaler et al. [42] were sampled only during the flight period and hence do not differ between the curves.

**Figure 2:**
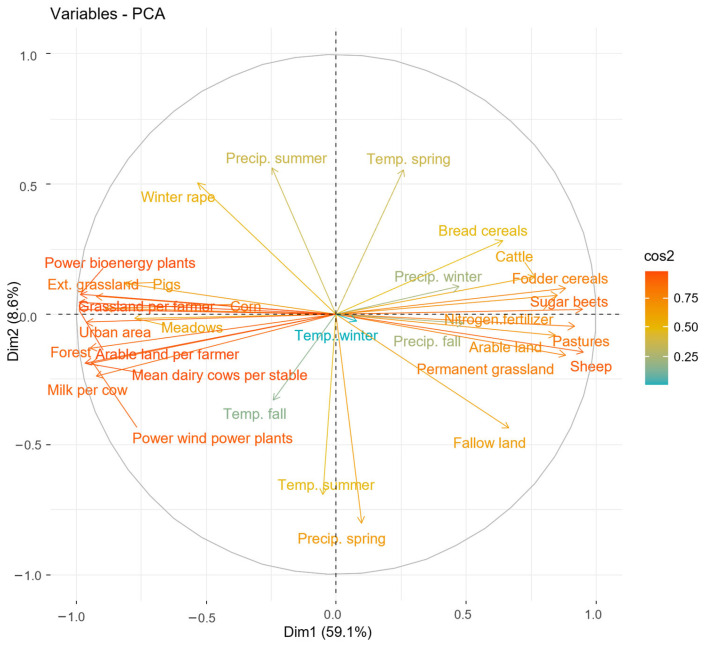
Biplot with visual representation of the relationships between variables for the full biomass dataset in the PCA. The direction of an arrow indicates the correlation between the variable and the principal components. The length of an arrow represents the variable’s contribution to the principal components. Longer arrows correspond to variables that contribute more to the variance explained by the principal components. Arrows pointing in the same direction are positively correlated, while arrows pointing in opposite directions are negatively correlated. The colors visualize the cosine square values (cos2), which indicate the quality of representation of that variable on the two depicted principal components. Arrows in red (high cos2 value) indicate that a large proportion of the variable’s variance is explained by the two principal components.

The dataset of sampled flying insect biomass from the study by Hallmann et al. [14] includes detailed information on the biomass of insects sampled over a defined period of days per sampling location (S1 Supplementary Material of Hallmann et al. [14]). As shown in Figure 2B of Hallmann et al. [14], the sampled insect biomass exhibited considerable variation throughout the year, with the greatest catches occurring during midsummer. Figure 2A in the study by Hallmann et al. [14] presented boxplots reflecting this high variability. To reduce the noise in the dataset, we standardized the data by focusing on the main flight period of insects, which roughly spans from 1 April to 30 September each year (as can be seen in Supplementary File S5, Figure S1). This approach excluded samplings with very little biomass, which occurred in some locations (4.5% biomass was excluded which was found at 11.6% sampling days of the original dataset) that were sampled, e.g., until December. A comparison of the transformed and the raw data can be found in Supplementary File S5, Figure S1. Afterwards, we calculated the mean daily biomass for each of the 57 sampling locations in NRW (Figure 1). For the following analyses, we used the mean value of those mean daily biomass figures to derive a single value per year. This standardization step offered the advantage of significantly reducing the dataset’s variability which originally included data not only from locations which were sampled only once, but which could even be assigned to different habitat types [14]. As an example, flying insect biomass (FIB) at four different locations sampled in 1994 varied between a mean of 3.04 g per day and 9.7 g per day, making it difficult to assign this variability to the outcome of certain pressures on insects or only to site and habitat effects. By using the annual mean values, we could achieve a more focused depiction of biomass trends and patterns over the study period.

In addition to the Hallmann et al. [14] data, we used the mean daily biomass data for the years 2020 and 2021 depicted in Table S1 of Mühlethaler et al. [42] for two sampling locations in NRW (Table S2 in Mühlethaler et al. [42]). In this study, flying insect biomass was sampled using Malaise traps, like in Hallmann et al. [14], between early April and the end of August and therewith within the main insect flight period.

Overall, the sampled flying insect biomass (FIB) decreased over time (Figure 1). The sampled FIB was rather high with a mean of 5 to 9 g per day with a high variation until the early 2000s. After 2007/2008, sampled FIB remained low until 2021. Note that the sites sampled in 2007 and 2008 were not sampled again. The high variation within a year, especially between 1989 and 1994, can be explained by the sampling of different habitat types and sampling durations. Although the data by Hallmann et al. [14] were normalized for this analysis to the main insect flight period of the year, the sampling duration had a major impact. For instance, the outlying 2011 data point was derived from a single sampling location during the time of the highest FIB occurrence from July to September, totaling only 67 days of sampling. In comparison, the data point of 1995 represents a single sampling site which was sampled over a 178-day period, covering months with lower insect activities as well.

### 2.6. Statistical Analysis

**Figure 3:**
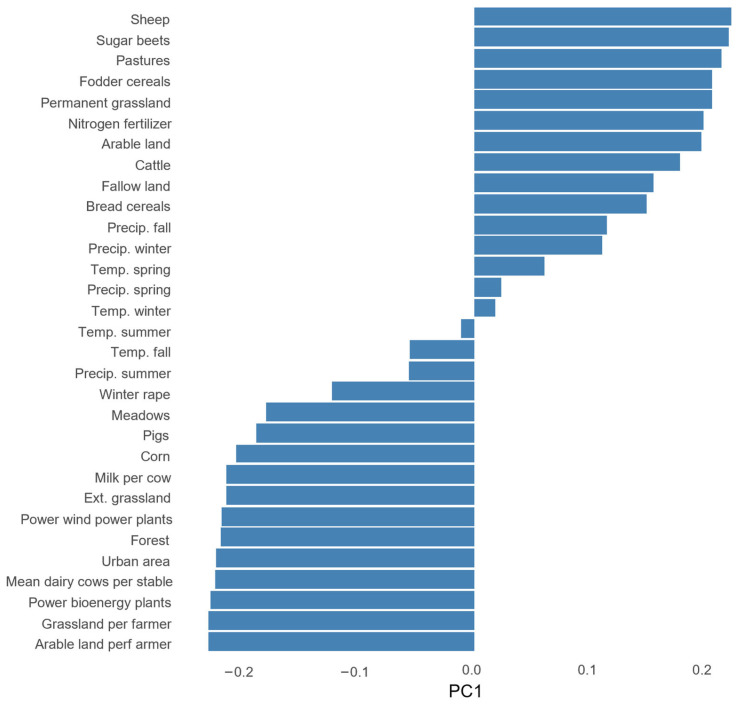
Depiction of variable loadings on PC1 for the full biomass dataset. The length of the bar represents the magnitude of the loading, and the direction indicates the sign of the correlation between the variable and PC1. Variables with higher absolute loadings contribute more to the variance explained by PC1.

A total of thirty-three predictor variables were selected from the land use and farming, renewable energy, pesticides, seed treatment, and weather data (see Figure 3 and Supplementary File S6 for a list and description of variables) to identify the most important variables explaining the decrease in FIB in the federal state of NRW.

For the statistical analysis, we averaged the insect biomass per day for all sites in each year to represent the average biomass per day at the landscape level. This FIB averaging resulted in 24 datapoints, one datapoint per year. Averaging biomass over each year was done for two reasons: first, as described earlier, the sampling design from Hallmann et al. [14] is lacking repeated sampling of most sampling locations which would be needed to detect reliable relations between predictors and FIB over time on smaller scales; second, for most predictor variables, a more granular time series is not possible or useful/meaningful, such as cropping statistics or urban area development, respectively.

Nine predictor variables contained missing values across the 24 years of biomass data (compare Figure 3). The number of missing values per variable ranged from 1 to 8. Excluding variables or years with missing values would have significantly reduced the available data for analysis, potentially impacting the power of the study. Therefore, spline imputation was chosen to address these missing values for seven of the nine variables.

Two predictor variables, toxic load of pesticides and seed treatment, exhibited non-linear trends through time (compare Supplementary File S1, Sheet 4 and Figure S3). Additionally, missing values were concentrated in the first few years (7 and 6 years, respectively). Imputing these values would not accurately reflect the true trends and could introduce bias.

Therefore, we adopted a dual approach:

1. Analysis with full biomass dataset and reduced variable set: We conducted the primary analysis using all 24 datapoints but excluding the two variables covering pesticides with non-linear trends and concentrated missing values. This ensured the inclusion of valuable long-term information while avoiding potential biases from imputation.
2. Sensitivity analysis with reduced biomass dataset and full variable set: To assess the potential impact of excluding these variables, we ran a secondary analysis including both variables but reducing the dataset by the first seven years. This analysis served as a sensitivity check and provided insights into the robustness of our findings.

Selecting appropriate methods for assessing variable importance is crucial when analyzing high-dimensional datasets with multicollinearity issues. A simple linear regression as a method for analyzing the effects between FIB and the predictors would have been problematic due to the multicollinearity between predictors and the low ratio of the number of datapoints to the number of predictors, which would lead to overfitting [61]. We employed Principal Component Analysis (PCA) to reduce the multicollinearity and dimensionality of the dataset. PCA was conducted using the ’prcomp’ function in R [62], allowing us to identify the principal components that explain the most variance in the data. A scree plot was generated to visualize the eigenvalues associated with each principal component, and we applied a threshold of an 80% cumulative proportion of variance to determine the number of components to retain for further analysis. Following this criterion, we selected the first four components for the full variable set and the first five for the reduced variable set, which accounted for a cumulative proportion of variance of 82.1% and 83.8%, respectively. We further calculated the cosine square values for each variable in the principal components. The cosine square values provide insight into the importance of each principal component by indicating the extent to which the original explanatory variables are represented within each component. High cosine square values suggest that a component effectively captures the variance associated with specific variables, thereby enhancing its relevance in subsequent analyses. The calculation was performed using the ‘factoextra’ package in R [63].

Subsequently, we utilized a linear model to assess the relationship between the selected principal components and FIB. Prior to fitting the model, we verified the assumptions of linear regression, which included linearity, homoscedasticity, normality of residuals, and multicollinearity. Linearity was examined by visually inspecting the relationship between the model’s fitted values and residuals. Homoscedasticity was assessed using the Breusch–Pagan test [64], implemented via the ’lmtest’ package in R [65]. The normality of residuals was evaluated using the Shapiro–Wilk test [66]. Although multicollinearity was not expected due to the use of principal components, we further assessed it using Variance Inflation Factors (VIF) calculated with the ’car’ package [67].

## 3. Results

### 3.1. Statistical Analysis

In the PCA with the full biomass dataset and reduced variable set, the first principal component (PC1) can be broadly described as an “agricultural intensification and land use change gradient” (Figure 2 and Figure 3). This component is characterized by high negative loadings for variables associated with intensified agricultural practices, such as tons of milk per cow, power of energy in plants, and arable land cultivated per farmer. Conversely, PC1 exhibits positive loadings for variables indicating extensive agricultural practices, including the area of pastures, permanent grassland, and the number of sheep. Further, the component also includes land uses, with negative loadings of increases in urban areas and forest and positive loadings of total arable land. Overall, the high cosine square values (see Supplementary File S6, Figure S1A) of variables with high absolute loadings in PC1 (Figure 3) demonstrate a strong representation of agricultural intensification and land use change in this component.

The second principal component PC2 was primarily influenced by climate variables, specifically temperature and precipitation in spring and summer. Temperature and precipitation exhibited opposite loadings within the same season (Figure 2). The cosine square values of PC2 are lower compared to the cosine square values of PC1 (see Supplementary File S6, Figure S1B); however, it still indicates a reasonably good presentation of the listed climate variables in this component.

The PC3 showed relatively low cosine values for most variables, indicating limited representation of any single variable. Significant and opposite loadings for “Cattle” and “Meadows” can be observed, but the overall weak representation suggests that PC3 may capture less relevant patterns in the data (see Supplementary File S6, Figure S1C). Similarly to PC2, PC4 and PC5 were primarily influenced by climate variables; however, the generally exhibited low cosine values (see Supplementary File S6, Figure S1D,E), indicating limited explanatory power and weak associations with the original variables.

The linear model analysis with the reduced variable dataset revealed significant relationships between FIB and the retained principal components derived from the PCA. Among the five principal components, only PC1, representing the “agricultural intensification and land use change gradient”, was identified as a significant positive predictor of FIB (Table 1). This indicates that higher values of PC1, which are associated with extensive agricultural practices, correlate with increased FIB.

In contrast, the other principal components (PC2, PC3, PC4, and PC5) did not demonstrate statistically significant relationships with FIB. PC3 was the only component which showed a trend, with higher values, associated with more meadows and cattle, positively correlated with FIB. The overall model demonstrated a multiple R-squared value of 0.69. A partial R-squared value of 0.66 for PC1 indicated that this principal component explained a substantial portion of the variance in FIB for this dataset.

In the sensitivity analysis with the full variable set, the PC1 is very similar to the PC1 calculated for the reduced variable set and can also broadly be described as an “agricultural intensification and land use change gradient”. Interestingly, the total toxic load of seed treatments in agricultural landscapes positively correlated with variables associated with extensive agriculture. As in the reduced variable set, the PC2 was also heavily influenced by climate variables. In contrast to the reduced variable set, however, the main contributor of the component was the newly added toxic load of pesticides in the agricultural landscape. The results of the linear model with the full variable set were similar to the results with the reduced variable set, i.e., only PC1 was a significant predictor of FIB, suggesting that higher values of this agricultural intensification and land use change gradient are associated with lower FIB. Interestingly, higher toxic loads of seed treatments were also linked to higher FIB. More detailed information regarding the sensitivity analysis can be found in Supplementary File S6.

### 3.2. Changes in Agriculture and Land Management over Time

**Figure 4:**
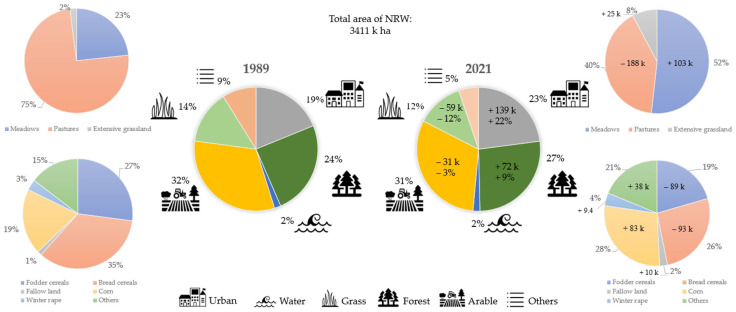
Comparison of the proportions of the main landscape variables, the different permanent grassland types and the main crop types between 1989 and 2021 in NRW. For 2021, the relative differences to 1989 are indicated in percent and/or in 1000 hectare.

Over the study period of 33 years, the overall landscape management and land utilization changed to varying degrees (Figure 4). Between 1989 and 2022, arable area decreased by 2.9% (−30.7 k ha) and permanent grassland by 13.6% (65.2 k ha), while urban area (which includes traffic areas) grew by 25% (+139 k ha, in total covering 24% of NRW) and forest cover increased by 9% (+71.5 k ha, Figure 4).

**Figure 5:**
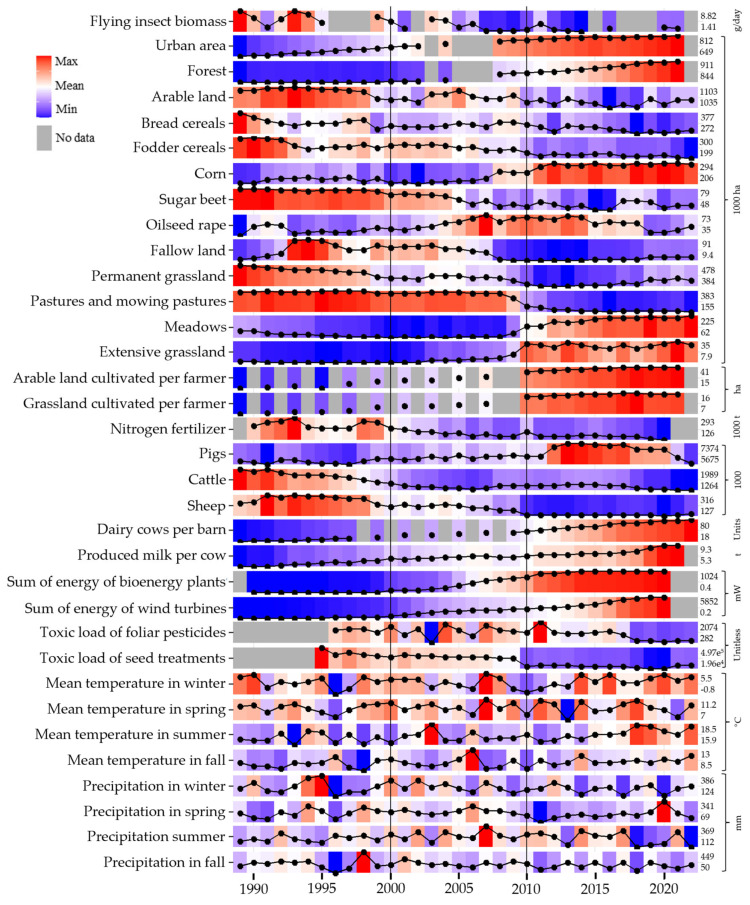
The complexity and visual correlation of potential drivers behind insect decline. The time series of selected variables spanning from 1989 to 2022 (where data was available) are depicted. Included in the presentation are the annual mean flying insect biomass (FIB), derived from Hallmann et al. [14] and Mühlethaler et al. [42], and various landscape, agriculture, renewable energy, climate-related variables and the toxic load of pesticides for the federal state of North Rhine-Westphalia (NRW) in Germany. Additionally, on the right side of the presentation, the minimum and maximum values for each variable are specified as well as vertical lines for visual aid. More detailed visualizations of the variables are presented in Supplementary File S5.

The share of arable land used for bread cereals (wheat and rye) dropped from 35% to 27%, and fodder cereals decreased distinctly from 27%to 19%. Corn cultivation, the third largest crop type, grew by 37% to 27% of the total arable land driven by silage corn after 2009 (Figure 5). Winter oilseed rape started to increase after 2004 with a peak in 2007 of 7% (Figure 5). A more detailed figure of the main crop types, including the yield per hectare and harvest of the most relevant crops, is provided in the Supplementary File S5, Figure S3B,C.

Fallow lands peaked at 8% in 1994, remained at high levels with 71.4 k ha until 2003, and declined sharply to below 10 k ha by 2012 (Figure 5).

The area of permanent grassland, which is land used permanently (i.e., for five consecutive years, or more) to grow perennial grasses and other plants which is not included in the crop rotation, decreased over time by 13.6% with two periods of change (Figure 4 and Figure 5). The first one was a slow and fluctuating decrease until 2008, whereas the second wave from 2008 to 2011 was distinctly more pronounced, corresponding to 45% of the total grassland loss (39 k of in total 86 k ha). The permanent grassland decline peaked in 2013 with a total loss of 93.8 k ha, corresponding to a 24.4% decrease compared to 1989. After 2013, it saw a slight recovery.

Extensive grasslands are grasslands which are non-intensive grazing pastures, but also included grasslands taken out of management. These increased after 2005 from 2% to 8% of permanent grassland area in 2013.

The second wave of permanent grassland decrease starting in 2008 was also the beginning of a pronounced transformation of grassland management from pastures to meadows. While in 2008 over 80% of the permanent grassland was managed as pastures and mowing pastures, their share was reduced to less than 38% in 2022 (Figure 4 and Figure 5).

While arable and grassland use changed, the arable and grassland cultivated per farmer increased distinctly.

Cattle numbers, including dairy cows, decreased constantly by about 25% by 2006/2007, and stabilized with dairy farming consolidation into fewer, larger barns (see Supplementary File S5, Figure S3G,H). In 1989, 85% of dairy cows were reared in barns with less than 50 animals and 99% in barns with less than 100 animals. In 2022 these numbers were down to 9.5% for barns with up to 49 animals and to 33.5% in barns with less than 100 animals (see Supplementary File S5, Figure S3H). At the same time, milk production per dairy cow increased by 48%.

Sheep numbers fell from roughly 300 k in the 1990s to roughly 220 k in the early 2000s followed by a drop after 2009 to 130 k. Conversely, the number of pigs consistently increased over time with a sudden increase in the year 2012 (Figure 5).

The tonnage of artificial fertilizer sold dropped significantly by 90%—with phosphate fertilizer down by 90% and nitrogen fertilizer down by 57%. Figures for organic-based fertilizing (such as manure, e.g., used for intensive grassland management) were not available.

Renewable energy generation rose sharply with two waves of bioenergy plant installations. The first wave started in 2004 and peaked in 2006 and the second wave started in 2009, peaked in 2011, and led to a substantial increase in electrical power generated with 293 new bioenergy plants generating an additional 16.6 megawatt of power generation (Figure 5).

Wind energy generation also grew, with a surge in plants between 2000 and 2005, and more powerful installations after 2011 (see Supplementary File S5, Figure S3I).

### 3.3. Pesticides

#### 3.3.1. Foliar Applications

**Figure 6:**
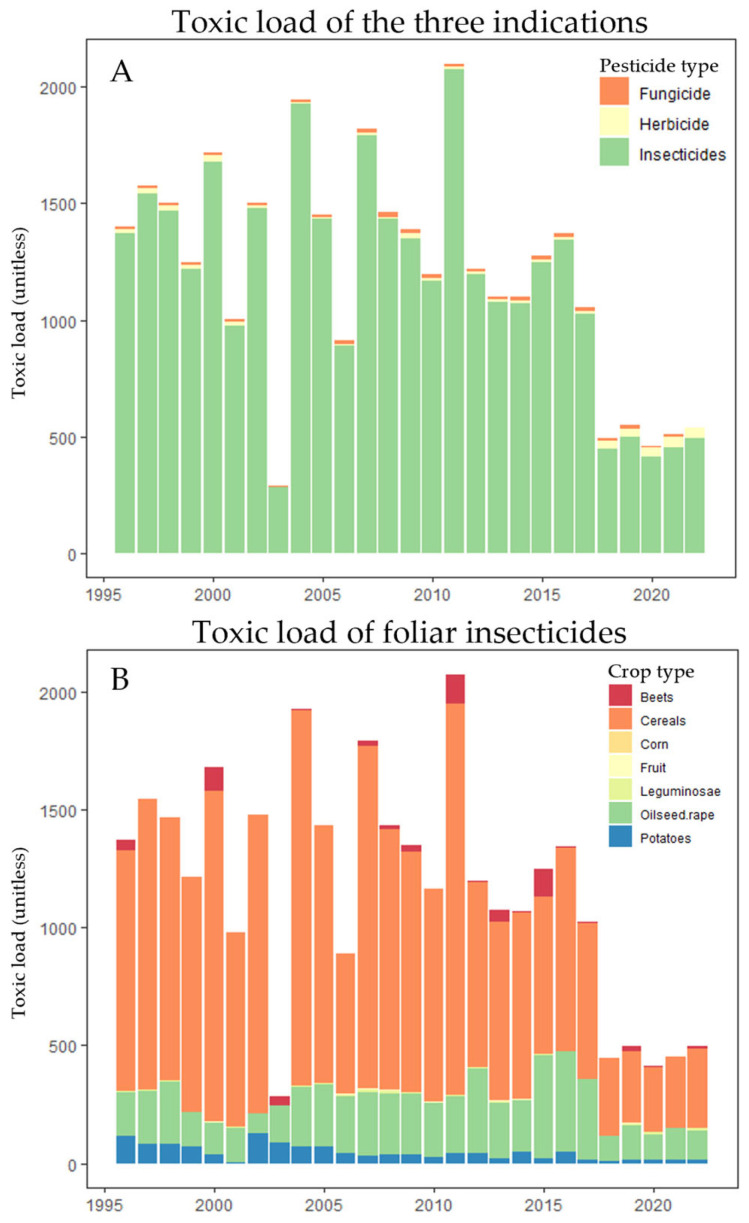
Toxic load of foliar-applied pesticides overall (**A**), and of foliar-applied insecticides per crop (**B**).

The overall toxic load of pesticide foliar applications fluctuated over the time period from 1996 to 2022 but showed a clear declining trend from the early 2000s (Figure 6), especially after 2017. Hence, the intrinsic toxicity of foliar-applied pesticides has been decreasing, as the overall cropping area has remained rather constant over time.

Of the applied pesticide tonnage calculated over the whole study period, 71.2% were herbicides, 28% were fungicides, and 0.8% were insecticides (Supplementary File S5, Figure S4A). However, insecticides represent an overall 97% of total toxic load (Figure 6A). In our approach, crops cultivated in large areas, which therewith correspond to large areas treated with pesticides (such as cereals), contribute to the overall toxic load to a higher extent than crops with smaller cultivated areas (Figure 6B). In addition, this approach reflects realistic pesticide use scenarios by taking into account that some crops are treated with more intrinsically toxic pesticides, or a higher number of applications compared to other crops.

#### 3.3.2. Seed Treatments

The overall toxic load and the applied tonnage of insecticidal seed treatments showed an overall declining trend over time (Supplementary File S5, Figure S4B–D). Overall, five main crops were grown from insecticide-treated seeds with 100% of the cultivated area assumed to be treated, namely sugar beets, corn, oilseed rape, potatoes, and cereals. It has to be emphasized again that the assumption of a 100% treated area is a conservative worst-case assumption which leads to a pronounced overestimate of the toxic load, at least in some of the crops.

The toxic load decreased on all crops with the most prominent changes in the crops with the highest cultivated area, namely cereals and corn. A drop in toxic load in cereals after 1995 can be explained by the reduced active ingredient (imidacloprid) content in a neonicotinoid product that had entered the market by then. The toxic load in cereals dropped again after 2009 when seed treatment with neonicotinoids was no longer used (Supplementary File S5, Figure S4C). The overall toxic load increased again in 2021 after a cypermethrin-containing product was registered. The calculated toxic load in corn increased after products containing imidacloprid were registered in 1997, and for clothianidin as well as thiamethoxam in 2003. Similar to cereals, the toxic load in corn dropped after 2009 when seed treatment with neonicotinoids was no longer used. In sugar beets (6% of arable land), the toxic load decreased distinctly after carbofuran was replaced by neonicotinoid-containing products starting in 1999 and the stop of carbofuran-containing products after 2006/2007.

### 3.4. Weather and Climate

Over the whole study duration, colder years (e.g., 1996) as well as very warm years (e.g., 2014 and 2018) occurred. But also, extreme weather events happened, such as in 2007 with precipitation during the summer exceeding the long-term mean precipitation by 58%.

All eight selected weather and climate variables showed no distinct trends over the years of the study duration besides an array of years with warmer winter temperatures after 2013, and among the warmest and dryest summer starting in 2018 (Figure 5).

## 4. Discussion

Using a comprehensive set of environmental and economic parameters, we evaluated the changes over time for multiple potential drivers of insect decline in a region where a well-documented decline of flying insect biomass (FIB) was observed.

There are some changes in the environmental factors over time which may have had a stronger influence on the long-term development of insect abundances than others. Among the most prominent changes there was a significant loss of permanent grassland and an intensification of the management of the remaining grassland. Another major change in arable land was a strongly increased corn growing area to cover the growing demand of livestock farmers and bioenergy plants. At the same time, a loss of potential insect habitats took place due to extensive urbanization.

### 4.1. Changes in Landscape and Land Use

Over the study period of 33 years, we observed extensive and significant changes in the overall land management as well as intensification of grassland and crop production in the study region. While agricultural intensification can lead to habitat loss, declining habitat diversity and habitat fragmentation [5,68], the most significant conversion in terms of surface area was attributed to increased urbanization. The increase in urban areas (including traffic areas) in NRW was roughly equivalent to the combined area loss of grassland and arable land during the same time period. However, we were unable to determine precisely how much grassland was transformed into arable land and how much into urban areas, as such detailed data is not readily available.

Although urbanization and the resulting increase in impervious surfaces is rarely listed as a major driver of insect or biodiversity decline, negative direct and indirect effects are well documented for multiple arthropod groups [37]. While some insect taxa, such as (wild) bees, can thrive in gardens of urban and suburban areas [69], urbanization negatively affects most arthropod communities in various ways: Habitat loss due to impervious surfaces of buildings or roads [70,71] as well as a significant increase in disturbances e.g., through leisure activities are examples of direct effects. At the same time, the remaining open spaces become more fragmented, making it more challenging for arthropods to move between suitable habitats [72]. Additionally gardening with non-native flowers can lead to reduced habitat quality [73].

Overall, urbanization has been found to generally decrease pollinator diversity and abundance [74] and lead to the homogenization of (mobile flying) insect communities [36,75,76], and generally, flying arthropod abundance is negatively associated with impervious surfaces [71].

The insect biomass samples by Hallmann et al. [14] and Mühlethaler et al. [42] were collected in different kinds of conservation areas in which habitat loss due to increasing urbanization will normally not have a direct effect. However, in the study region, conservation areas are often very limited in size and embedded in agricultural or urban landscapes. Hence, urbanization in surrounding areas can have far-reaching effects on flying insects which potentially impact the communities in the sampled conservation areas. For example, Concepción et al. [77] highlight the harmful impact of widespread urban expansion on organisms, such as butterflies, which avoid urban areas, and that highly mobile and specialized species are at especially risk. Piano et al. [37] showed that actively dispersing species declined drastically in response to urbanization.

Recent studies which analyzed the effects of landscape factors on flying insect biomass support our evaluation of the negative effects of urbanization. Uhler et al. [15] compared flying insect biomass between different land use types including agricultural areas but found the strongest decrease in insect biomass of 42% between semi-natural and urban environments in southern Germany. Svennigsen et al. [78] found a negative association between urban cover and flying insect biomass in Denmark and Germany using rooftop-mounted car nets at a landscape scale.

While it can be expected that the impact of transforming natural or seminatural habitats into urban areas has strong effects on insect biomass and biodiversity, the urbanization of arable land can likewise have a wide-reaching impact. Wan et al. [79], for instance, showed that increasing urbanization in China decreased populations of abundant pest moth species, especially where agricultural land was lost. Such a loss of abundant pests might be detectable in flying insect biomass samples.

One of the indirect effects of urbanization, which is becoming apparent in rural or even conservation areas, is artificial light at night (ALAN) that is emitted by buildings and streetlights [80]. ALAN, also known as light pollution, has been addressed as an important direct driver of insect decline (described in Grubisic et al. [81]). More than 60% of invertebrates are estimated to be nocturnal [82], including 75–85% of Lepidoptera [83]. Flying nocturnal insects often show flight-to-light behavior like many moths [84] and are hence attracted to light. Since the sampling design of Hallmann et al. [14] focused on flying insects, increasing ALAN intensities over time could have been partially responsible for reducing sampled insect biomass, since nocturnal flying insects are potentially confused, deterred, or lured depending on their behavior [80]. Such a direct effect on nocturnal flying insects is difficult to assess on a federal state level, as ALAN particularly occurs in urban areas which are not evenly distributed in the landscape. A case study addressing this topic and also covering an area around sampling points of Hallmann et al. [14] is the study by LaRoe et al. [80].

Like urban areas, forest cover increased over the duration of the study in NRW by 8.5%. Increased forest cover was also found by Hallmann et al. [14] in proximity to their sampling sites. Unfortunately, no information is available on which types of areas were transformed into forests over time although the transformation of grassland to forest is more likely than arable land to forests due to the higher profitability of arable land compared to grassland. Likewise, no specific information on forest management, which can have an immense impact on arthropod communities, is available [85]. The potential impact of increasing forest cover on the sampled flying insect biomass is therefore difficult to assess. In a comprehensive study, Seibold et al. [85] showed arthropod decline in forests as well and therewith demonstrated that loss is not restricted to open habitats. Meanwhile, Uhler et al. [15] sampled different landscapes across southern Germany and found that biomass was highest for forests and significantly lower for arable fields followed by urban areas.

Increased forest cover could lead to shifts in insect community compositions, although this might not be evident in the insect biomass data. In any case, effects of forest cover on flying insect biomass have been shown not to be necessarily positive: Blumgart et al. [86] found a significant decline in moth biomass and abundance in broadleaf woodlands surpassing the decline seen in intensively managed farmland. They were not able to detect the drivers behind the decline but showed that moths did not benefit from an increase in woodland habitats. In addition, Welti et al. [39] showed that, across Germany, flying insect biomass declined with increasing forest cover in the area surrounding the trap locations. They explained this as reduced floral resource availability compared to open fields [87] but also that forest vegetation structure may limit insect movement through the landscape, reducing the trap catch compared to open systems like grasslands [88].

Many major changes in central European landscapes can be attributed to the implications of EU policies [89,90], such as the observed afforestation which was strongly supported by the EU (Regulation 2080/92) or later as an answer to climate change [91]. Another example for comprehensive landscape changes or habitat loss driven by political and economic reasons is the development of fallow land in NRW. Fallow lands, arable land taken out of management, are known to harbor high plant and animal diversities which can be distinct compared to other habitat types [92,93]. Fallow land or set-aside land areas fluctuated greatly over the time of this study. Initially, it started as a voluntary measure to increase the competitiveness of European agriculture. Later, it became an obligatory policy instrument stipulated in 1992 (MacSharry Reform) to limit harvest surpluses [94]. Farmers received area-based subsidies for areas which were taken out of management [95,96]. As a result, at its peak in 1994, fallow land covered more than 8% of the total arable land in NRW. However, the areas targeted by the EU policy for fallow land in the EU decreased over the years from 15% in 1992 to 5% in 2004/2005, and the fallow land obligation in the EU for 2008/2009 was initially suspended [97] due to increasing prices for agricultural commodities. As a result of these changes in EU agricultural policies, a significant amount of fallow land roughly equal to the area of all pastures in NRW was lost between 2003 and 2008. The loss of extensive areas of high-diversity habitats and the resulting simplification of the landscape can have far-reaching consequences for insect populations in these areas [92].

Though fallow land was not necessarily taken out of management, after 2004 EU policies allowed farmers to grow non-food crops on set-aside land. This shift aimed to support renewable energy production, with crops such as oilseed rape for biodiesel and corn for biogas plants becoming prominent. The cultivated area for oilseed rape increased by approximately 50% since the early 2000s, peaking in 2007 due to rising demand for biodiesel under EU renewable energy policies. Consequently, the total area of fallow land declined, and a significant portion of remaining set-aside areas was repurposed for energy crop production.

This transition likely had notable consequences for insect populations. Fallow land often serves as a refuge for insects, offering diverse floral resources and nesting sites [98,99]. Replacing these high-diversity areas with monocultures like oilseed rape or corn reduces habitat heterogeneity and resource availability, e.g., via herbicide usage [100,101].

### 4.2. Agricultural Intensification

Agricultural intensification is defined [102] as the process of increasing the use of capital and labor (e.g., fertilizers, pesticides, machinery) relative to land area, to increase agriculture production per hectare. It has been reported to be a primary driver of insect and overall biodiversity decline [30,38,103,104], although the effects might be less severe for insect biomass than for species richness [15,21]. A potential explanation for the latter effect is that pest species and other species adapted to agricultural habitats, such as aphids, thrive in agricultural landscapes and can contribute significantly to sampled flying insect biomass [21].

Unfortunately, agricultural intensification on a local landscape level, such as a reduction of hedgerows, loss of vegetated field edges, lacking connectivity of habitats, or the consolidation of arable fields and other direct effects on insects, cannot be analyzed with the available datasets. Such information is complex and is neither available on larger scales such as federal states, nor systematically and consistently available on a local level.

As a proxy of agricultural intensification, an increase in yield per hectare can be seen, which can be due to either improved crop varieties, changes in fertilizer or pesticides, or other crop management practices. For cereals in general, sugar beets, potatoes, and oilseed rape, yields increased by roughly 40% to 50% over the three decades. With this yield increase, a rather constant harvest could be ensured while the area cultivated with these crops partly decreased (Supplementary File S5, Figure S3C).

Increasing nitrogen applications in grasslands are often in the list of biodiversity decline drivers [105] but affect insects only indirectly via the homogenization of plant communities [106,107,108,109], which often correlate with insect diversity and biomass [110,111].

Such an increasing nitrogen application could not be derived from the sold tonnage of artificial nitrogen and phosphate fertilizers, which has decreased by over 50% over time. It is most likely that artificial fertilizer was applied in the regions where it was sold due to costs for transport; nevertheless, the actually applied tonnage might differ. More importantly, the input of organic fertilizers, such as liquid manure, could not be quantified, but it is likely to have increased in during course of intensified animal husbandry.

This study could not consider the quantities of organic fertilizer, such as biogas fermentation residues and manure, or slurry from cattle or pigs, due to the unavailability of reliable data. Although the number of cattle decreased over time, it is unlikely to have resulted in decreased manure spreading on grassland or arable land. The contrary may be the case due to a shift from pasture to barn feeding [112]. Most likely as a result of increasing exports to non-EU countries [113], the number of pigs increased distinctly in NRW and in the Netherlands which is (together with Denmark) the biggest pig meat exporter in Europe. According to Willems et al. [114], in 2012, 60% of exported Dutch manure and slurry went to Germany, mainly in NRW which shares a border with the Netherlands. The contribution of imported manure from the Netherlands on applied nitrogen in the environment is also highlighted by Häußermann et al. [115]. So, the recorded decrease in sales of artificial fertilizer may even be explained by an increased use of organic fertilizer, so that the overall quantity of applied fertilizer might even have increased rather than decreased over the last decades.

A side effect of the direct application of inorganic and organic nitrogen-rich/ammonium-based fertilizers is the emission of nitrous oxide which can affect not only adjacent areas but also areas which are further apart. The accumulation of atmospheric nitrogen results in the eutrophication of soils which leads to a reduction in plant diversity [106,116].

Another potentially important indicator of agricultural intensification is the size of the mean arable land cultivated by each farmer which increased by 170% over the study duration as a result of a substantial decrease in agricultural operators of 65%. While some farm exits can be attributed to retirement [117], the main mechanisms behind the decline of farmers identified by Strijker [118] is the change in relative prices of inputs and outputs. According to Strijker [118], there was a rapid increase in the opportunity costs of labor which led to the mechanization and intensification of agriculture which has further been stimulated by the Common Agricultural Policy [119] of the EU and policy developments. The increasing size of the arable land cultivated per operation can be seen as a proxy of agricultural intensification and landscape homogenization, as it most probably is accompanied by overall larger field sizes.

Farmland consolidation often involves the removal of field boundaries and hedgerows to create larger, more homogenous fields. While these changes improve economic performance by reducing labor costs and enabling mechanized farming [114], they significantly alter the landscape. Larger, simpler fields lack the structural complexity provided by hedgerows and field margins, which serve as crucial habitats for insects [106,115]. The removal of these boundaries and therewith the simplification of landscapes also reduces corridors for movement, further isolating insect populations and exacerbating declines in biodiversity and ecosystem function [104,120].

### 4.3. Grassland Intensification and Livestock Farming

As mentioned above, the area of permanent grassland decreased over time and the habitat loss associated with these changes has been considered to be one of the direct effects on insects and drivers behind insect decline [17,121]. Together with the decrease in grassland, the management and therewith the habitat quality for its inhabitants also changed, which in the case of intensification can have negative effects on arthropods [107,122,123]. Similar to arable land, the size of the mean permanent grassland area managed per farmer increased over time. Larger grassland plots can be assumed to indicate management intensification as, e.g., it can be managed with larger machinery. Another aspect of grassland management which could not be analyzed with the available dataset is sowing seeds, e.g., of feed crops in permanent grasslands. This practice involves disturbing the topsoil (e.g., milling), followed by grassland reseeding and, depending on the used seed, can enhance the nutritional value of forage for livestock, increasing productivity by promoting the growth of more productive plant varieties, controlling weeds, but also by promoting biodiversity [124,125,126]. A potential intensification of grassland management cannot be shown but is likely in terms of an increasing overall management intensity as described below. The increase in grassland yield could be also seen in the distinct increase in field grass area (see Supplementary File S5, Figure S3E) between 2008 and 2013, the years with a strong decrease in permanent grassland (see Supplementary File S5, Figure S3D). This change suggests that parts of permanent grassland were plowed up and hence transformed into arable land to cultivate grass.

Another aspect of grassland management intensification is the development of extensive grassland areas. In our analyzed dataset, extensive grasslands include non-intensive grazing pastures but also grasslands which have been taken out of management. This encompasses habitats with high diversities, such as dry grass meadows, as well as abandoned grasslands facing or undergoing shrub encroachment [127,128,129]; in so far as there are very different habitat types conflated under the term “extensive grasslands”, the influence on insect abundance and biodiversity may be variable or even opposed. Unfortunately, the available data do not allow for further specification and a separation of both types. This leads to the seemingly counterintuitive finding that extensive grasslands increase along with the decline in FIB.

The strong increase in extensive grasslands starting in 2005 may be attributed to a shift in subsidies for farmers as a result of the cross-compliance approach of the European Union’s Common Agricultural Policy (CAP) of 2003. As a part of this, Germany transitioned to fully decoupled payments and to an area-based single farm payment (SFP) approach. With this, the EU encouraged farmers to adopt sustainable agricultural practices and to focus less on maximizing production [130]. However, this shift in the payment policies may have had some impact on land management practices [131]. Without production-related subsidies, farmers might have reduced the size of grazing livestock herds and a decrease in grazing livestock herds can lead to surplus grassland that is no longer needed for grazing [132]. This grassland was either abandoned or converted into arable land, especially where there was a high demand for certain crops. Moreover, after another reform of subsidy payments in 2010, receiving subsidies for extensively managed grasslands became more challenging [133,134]. Most likely, grasslands with low profitability were abandoned and therewith taken out of management. While this set-aside grassland can promote insect diversity for some years [98], in the long run, it likely had direct negative effect for grassland insect biodiversity [135], which is fostered by extensive management, such as temporary grazing [92].

The period of substantial grassland decrease starting in 2008 was likewise the beginning of a comprehensive transformation of grassland management towards a higher management intensity. Pastures and mowing pastures were transformed into meadows.

Data on pastures which are not subject to mowing is available only between 1989 and 2008 and show a decline of over 50%. Hence, an ongoing transformation of grazing pastures to mowing pastures (as well as the number of grass harvesting events per year on those pastures) can be assumed after 2008 as well. In addition, the sudden transformation to meadows led to an increase in total grass harvest, but the overall yield per hectare meadow was halved. Therefore, it must be assumed that the created meadows were of low quality for both hay and silage grass growing.

Additionally, the change from pastures to meadows indicates a strong reduction in livestock grazing activities, independent of the grazing duration or livestock type. The change of management type can be beneficial for arthropods if it results in management extensification. Studies by, e.g., Tälle et al. [136] or Török et al. [137] show that a reduction in grassland management intensity is beneficial for arthropods, independently of which management parameter (number of mowing events, reduced grazing intensity) is the subject of extensification.

In a study by Laggner et al. [138], the permanent grassland management in Lower Saxony, the adjoining federal state to NRW, is reported for the year 2005. There, 60% of permanent grasslands were managed by farmers with dairy farming and 25% used for other cattle, sheep, and horses. This suggests that grassland management is driven by livestock farming and changes in livestock farming will have direct effects on the ecosystem of permanent grassland and its biodiversity [139,140,141]. Over the study period, the number of cattle, dairy cows, and sheep decreased, which, however, came along with a management intensification of grassland. In NRW, the number of sheep on pastures decreased constantly in a similar way to permanent grassland, with a distinct reduction between 1998 and 2000, and between 2008 and 2010, the time period with a high transition rate of pastures to meadows. This is a strong indicator of grassland intensification as the profitability of sheep is very low and hay or silage grass production results in a higher monetary output.

Overall, as sheep pastures harbor high insect and plant diversities [141,142], they are an indicator for low-intensity pasture management and a declining number of sheep indicates a decline in grassland of low management intensity.

As roughly 60% of permanent grassland is managed for dairy farming, changes in dairy farming will impact grassland management. We could show that, over the study period, dairy farming has changed considerably. Traditional dairy farming with pasture feeding is decreasing and is being replaced by intensive dairy farming, as can be seen by the increasing barn size or in the amounts of produced milk per dairy cow, which increased by 48% over the 33-year study period.

The intensification of dairy farming with permanent animal housing in barns reduces the demand for grazing and the increased milk production requires new cattle breeds and nutritionally balanced diets containing higher amounts of grain corn and corn silage (up to 67%) and grass silage which is harvested from meadows [143,144]. The reduced or absent pasture feeding of cattle on grasslands can have substantial direct effects on arthropod abundance [141]. It has been shown that flying insects are significantly more abundant on grazed pastures compared to silage meadows [145], in particular at times when dairy cows are present [146]. This is most likely due to the availability of cattle feces which provide microhabitats for hundreds of mostly flying insect species to reproduce and feed on [147,148,149], depicting a key component of arthropod diversity in grassland ecosystems. It can be estimated that one pasture-fed cow is “equivalent” to at least some hundred kg insects per season which feed on or develop in its feces (according to information, e.g., from [147,148,150,151]).

While silage corn and grass silage are used to feed dairy cows, grain corn is used as feed for dairy cattle, beef cattle, and pigs. The significant steady increase in grain corn over the study period can be explained by a higher demand of feed for livestock.

The development of grassland and livestock management is a good example highlighting how interconnected and interwoven factors are that are associated with and that likely lead to a decline in insect populations: more intensive cattle breeding goes along with increased barn feeding; this, on the one hand, leads to a growing demand for feed which is produced on intensively managed grassland or through the cultivation of silage crops, both of which are likely to be associated with reduced insect abundance and diversity. On the other hand, barn feeding produces more liquid manure, which again reinforces grassland intensification due to the higher organic fertilizer input.

### 4.4. Renewable Energy

The intensification of dairy farming was a consistent trend but does not alone explain the distinct transformation of pastures to meadows and the loss of permanent grassland after 2008. The decline of grassland can also be considered a result of the second amendment of the renewable energy law (EEG) in 2009 [152,153,154,155], which caused an increase in bioenergy plants which had its peak in 2011. The EEG included a bonus system for the usage of renewable substrates like manure or energy crops [154]. An earlier amendment of the EEG started a smaller wave of bioenergy plant establishment in 2004 which resulted in an increase in bioenergy crops, mainly silage corn, but did not affect the grasslands. Laggner et al. [138] indicate that, until 2007, the feedstock needed to produce bio-methane has primarily been cultivated on existing arable land.

After 2008 the bioenergy plants increased in number as well as in power output, and hence in size [155]. The resulting increased feedstock demand could presumably not be covered by manure, since neither the number of cattle nor pigs increased distinctly in this period. The demand was hence covered by plants that are harvested green, mainly silage corn or field grass.

The area used for silage corn cultivation and plants that are harvested green increased in waves similar to the bioenergy plant establishment with the first starting in 2004, followed by a second major increase until 2012, the end of the second wave of bioenergy plant establishment. As a result, the generated power of bioenergy plants and the area of plants that are harvested green increased in a concurrent way.

After 2011, the transformation of permanent grassland into arable land was no longer permitted by law and farmers had to apply to be allowed to plow up grassland. This change was stipulated after a significant quantity of grassland was converted to grow silage corn (“Corning”) [154,156]. Hence, the loss of permanent grasslands between 2008 and 2011 is mainly due to silage corn production as a result of the EEG mandate. Similar observations are reported in the federal state of Schleswig-Holstein in Northern Germany [157] or Hesse in central Germany [156].

The subsidization of renewable energy crops like corn led to an increase in the costs of farming products. Arable land formerly managed for the cultivation of cereals or livestock feed were then used for corn production [158]. The subsidization of energy crops resulted in increasing rental fees for farmland [159]. Subsequently, areas with formerly low yield, like grasslands, were transformed into fields with a higher value yield.

However, grassland was not only transformed into arable land, as grassland management also changed simultaneously [44]. Grasslands that were no longer used for livestock could supply feedstock for regional biogas production [160]. In general, permanent grasslands present considerable potential for biogas production [161,162]. Hence, grassland used for silage grass production will be managed at high intensities to maintain a high feedstock yield. High grassland management intensities imply a high number of mowing events per season as well as nitrogen input, e.g., through fertilizer applications, such as digested residues from bioenergy plants. Both developments have negative effects on arthropods, as high intensities of both support fast-growing, dominant grass species. These grass species outcompete other plants, resulting in a decrease in plant diversity [163] and the accompanying decrease in various insect groups [31,99,123].

Overall, Germany’s renewable energy strategy and its implementation had an impact on arable land use and came at the expense of grassland as well as fallow and abandoned land [164]. The impact of the EEG will continue as the maximum percentage of corn as feedstock for bioenergy plants decreased by law from 60% in 2009 to 40% in 2023, with 30% planned by 2028 [165]. With this limitation, together with the decreasing number of farmed pigs and cattle producing manure, the feedstock for bioenergy plants will shift more towards alternative energy sources such as grass or leguminous plants. Immerzeel et al. [166] and Dauber and Bolte [167] review and discuss the positive and negative effects of bioenergy on biodiversity.

Similar to the bioenergy of plants, the sum of power generated by wind turbines and the number of wind power plants as well as the generated electrical power increased over the study duration. In fact, according to the report of Trieb [168], whose model indicates a strong impact of wind parks on flying insects, wind power plants were discussed as a threat to flying insects [169] which is overall currently a topic of controversial discussion [33]. However, empirical data by Trusch et al. [170] indicate a rather low impact of wind power stations on flying insects. These contrasting results suggest that the actual impact of wind turbines on FIB may not be straightforward to assess, and some insect groups, such as migratory species, may be more affected than non-migratory species [33]. On a very local scale, wind turbine aggregations could have a certain impact on insect abundance, but the likelihood appears limited that wind turbines directly cause the observed insect decline tendencies over large regions. The effect is likely to be less pronounced compared to the indirect effect of bioenergy plants, which influence large areas in terms of bioenergy crop cultivation.

### 4.5. Pesticides

Pesticides are frequently discussed as one potential driver of insect decline, especially in agricultural landscapes [171]. Indeed, the assumption of a driving role of pesticides, especially insecticides through direct and herbicides through indirect effects, appears intuitive, since insecticides are designed to kill insects, and herbicides to control weeds in agricultural crops which may serve as food sources for many insects. However, unlike other agricultural factors that may impact insect populations (e.g., structural changes, fertilization, plowing, harvesting), pesticides are subject to a rigid and meticulous environmental safety assessment in the context of the pesticide registration processes in the EU [172,173], which have been implemented to prevent unacceptable effects to non-target organisms in the environment. In the 1990s and early 2000s, risk assessment schemes for non-target arthropods in pesticide registration were revised and tightened, which led to improved environmental safety of crop protection products [174]. During the same period, newer pesticide products were developed that were based on more targeted mechanisms of action. These developments are reflected in the finding that the overall toxic load of pesticides has been decreasing over the years, as well as a lack of a correlation with declining FIB.

Pesticides as an actual pressure explaining insect decline have rarely been directly examined, though they are widely presumed to play a role [30]. A factor which makes it challenging to study potential correlations between the application of pesticides and the abundance of insect populations is the fact that detailed pesticide use data are not readily available in most countries. To overcome this limitation, other studies (e.g., [175]) have made use of sales data, which are more readily available (e.g., BVL [176]). However, sales figures have the disadvantage that they do not indicate when and where a product has in fact been applied, and thus only to a limited extent allow any estimates about spatial and temporal exposure of environmental organisms. In addition, the BVL [176] sales data are only available on national but not on the federal state level.

The limitation of data availability particularly applies to data with a high spatial resolution for the use of specific pesticide products. The only reliable data sources are commercially available market research data on pesticide use obtained from surveys among farmers. The lowest reasonably possible level of resolution for the analysis of this dataset was the federal state. Smaller scales, such as counties or districts, could not be reasonably resolved due to the limited number of data points per each of these units, which would have led to a pronounced influence of random effects or survey artifacts on the datasets.

The toxic load, determined as the calculated applied tonnage of active substance divided by the most conservative regulatory endpoint available, of foliar insecticides has shown an overall declining trend after the year 2000, which can be attributed to tightened regulations for the environmental safety of pesticides in the product registration process [177]. Changes in toxic load can also be influenced by changes in the area of cultivation of the respective crops. For instance, the total toxic load associated with oilseed rape increased after 2003 due to a rise in its cultivation for biofuel production. On the other hand, the toxic load of insecticides associated with cereals decreased by approximately 65% during the study period despite an only 28% decline in the cultivated crop area of cereals. Moreover, transient, erratic increases in the toxic load in certain years can be caused by increased pesticide application as a response to pest outbreaks in specific crops.

In addition to insecticides, herbicides are implicated in indirect effects on insects, such as reducing food availability for specialized herbivorous species or limiting flower availability in arable fields [178].

The applied tonnage of herbicides over time developed in accordance with the growing area of the respectively treated crops but overall remained on the same level (see Supplementary File S5, Figure S4A,F). Hence, the applied herbicide tonnage can be seen as a proxy for the growing area of the treated crops. As an example, the applied herbicide tonnage in corn increased similar to the corn growing area while in cereals, the crop with the largest growing area in the study region, the applied tonnage of herbicides decreased with a decreasing cropping area.

Our approach has significant strength in providing realistic pesticide exposure estimates, as it accounts in a differentiated way for scenarios where: (1) different crops are treated with pesticides that have different intrinsic toxicities; (2) crops are treated with different numbers of applications compared to other crops; or (3) the area planted with different crops is changing. This allows for a more accurate representation of the overall exposure of the environment to pesticides under consideration of their toxicity profiles. An example is the case of oilseed rape, even though it only covers 5% of the arable land in NRW, it contributes to ~20% of the total toxic load of insecticides. Similarly, orchards, which represent approximately <1% of arable land, contribute to 7% of insecticides applied (tonnage), but only 1% of the total toxic load.

An alternative model to evaluate pesticide risk potentials is the SYNOPS trend analysis [179]. In contrast to our approach, the SYNOPS trend analysis uses sales quantities of active substances, and the percentage number of uses registered for the pesticide in the different crops for each year as main input variables for the application scheme in the database. As a result, crops with small areas of cultivation but comparatively high insecticide applications, such as fruits, can represent a large part of the toxic load. On the other hand, crops with large areas of cultivation are comparatively underrepresented. Furthermore, several products are used by farmers almost exclusively in one crop, whereas the product is registered in other crops, too. Therefore, compared to the percentage-based number of all registered uses, our practice-based application scheme leads to more realistic results. Another advantage of our approach is that the greater accuracy of pesticide use data vs. sales data facilitates the identification of other factors which can be assumed to have influenced the use of pesticides, e.g., weather and climate. As an example, the drought in 2003 [180] led to a reduced need for insecticide application in cereals, but also in other crops (Figure 6B). Farmers typically use foliar insecticides as a curative application to reduce pest pressure in their fields. Other examples of the impact of weather on insecticide applications are from 2006, in which a very wet spring was followed by a very dry summer and a pronounced drought in 2018 [180]. In both years, insecticide applications were distinctly below the mean application schemes of other years (Figure 6B).

The SYNOPS trend analysis comes to similar findings as our toxic load calculation, and likewise shows a declining trend in the risk potential for non-target arthropods by pesticide uses to a comparable extent [181].

Seed treatments are primarily designed to protect seeds and young seedlings from pests, offering early growth stage protection when the plants are most vulnerable. The active ingredients in seed treatment pesticides are frequently systemic, meaning they are taken up by the plant and provide protection in the early growth stages. Seed treatments with insecticides are a targeted control measure against soil-dwelling and early-stage pests, such as chewing and sucking insects that directly attack seeds and seedlings.

The toxic load from insecticidal seed treatment products decreased between 1995 and 2006, when carbofuran, belonging to the carbamates, in seed treatments in sugar beet was taken off the market. However, neonicotinoids were the most prevalent insecticidal seed treatments until imidacloprid, thiamethoxam, and clothianidin were restricted (2008 [182,183] in Germany and 2013 in the EU) and eventually prohibited (2018) for outdoor uses in the EU [184,185,186]. These regulatory restrictions led to a drop in the overall toxic load of seed treatment after 2009 and 2018 (Supplementary File S5, Figure S4C). After 2018, only a reduced set of seed treatment products were available in agriculture (Supplementary File S3). Neonicotinoids, especially as seed treatment uses, have been controversially discussed over decades regarding their environmental safety [187,188,189] and have also been implicated in insect decline [190].

Other studies have employed scientifically more robust approaches that may pro-vide different perspectives. In cases where their results appear to contrast with ours, these differences may, in part, be explained by the methodologies used. For example, in some studies [51,171,191,192,193], the role of pesticides as a major driver is inferred from considerations of intrinsic toxicity rather than direct evidence. However, these approaches may overlook the fact that the environmental risk of pesticides is influenced not only by toxicity but also by exposure levels. Additionally, some studies apply correlation-based approaches that are similar to ours [194,195,196]. However, when the range of environmental variables analyzed is limited, there is a possibility that factors found to be associated with decline may not be causally linked, but rather act as proxies for other unexamined factors [197]. In this context, pesticide use might serve as a proxy for other factors related to agricultural intensification, which often occur together and can be challenging to disentangle.

### 4.6. Weather and Climate

In addition to the various factors discussed, weather and climate can play a critical role in shaping insect populations. Extreme weather events, such as the droughts in 2003 and 2018, can exert substantial impacts on insect abundance and diversity [35,198].

For example, Palmer et al. [199] observed that while some butterfly species exhibit sharp population changes in climatically extreme years, others remain unaffected. Similarly, prolonged dry and hot summers can drive rapid population declines in butterflies [200], grassland grasshoppers [201], and broader arthropod abundance [39]. Welti et al. [39] demonstrated that flying insect biomass drops significantly during summer months when long-term temperature averages are exceeded, indicating a direct link between temperature anomalies and insect declines.

Droughts, such as the one in 2003 characterized by rather low precipitation in February and March, followed by an unusually dry summer [202], can disrupt arthropod communities for extended periods. Frampton et al. [203] showed that such events can alter arthropod assemblages for at least 97 days. These disruptions can compound the effects of already degraded habitats, reducing the resilience of insect populations. This linkage between weather extremes, habitat quality, and flying insect biomass decline was further supported by Sohlström et al. [204], who found that land-use intensity exacerbates the negative effects of climate change on insect populations.

Although arthropod abundance may recover within a year after an extreme event [35], persistent weather anomalies, such as unusually mild winters or consecutive hot summers, could contribute to fluctuations in population dynamics and species composition [41,205]. For instance, Shan et al. [60] demonstrated that extreme weather events affect butterfly populations irrespective of the season, underscoring the pervasive impact of such events. Long-term climate change, particularly trends toward warmer and drier summers, is expected to have increasingly significant effects on temperate insect species in the future [206]. The ongoing warming trend in NRW, particularly noticeable after 2018, may already be signaling such a shift.

None of the selected weather parameters for which effects on arthropods were found by recent studies [14,16,41] appeared to be a relevant explanatory factor for FIB decline in NRW. This does not mean that weather and climate would not impact insects and their abundance; however, the lack of correlation highlights the complexity of climate impacts on insects [197].

It suggests that while climate and weather parameters significantly influence short-term fluctuations and species-specific dynamics, they may not be the primary drivers of the observed long-term trends in FIB decline. Instead, these factors likely interact with other drivers, such as habitat degradation and land-use intensification, amplifying their effects and contributing to the broader patterns of insect decline.

### 4.7. Limitations of Research

Although we are confident that our research approach can improve the understanding of how different environmental factors potentially impact insect abundance, population development, and diversity, this study encounters several limitations that should be acknowledged.

1. The most significant limitation is the heterogeneity and the lack of completeness of some of the long-term datasets. Although we widely used data which had an appropriate level of standardization and consistency over the years, some of the evaluated datasets still had gaps and limitations in consistency. For instance, pesticide data were only available from 1996 onwards and were generated according to a non-systematic scheme, or certain data terms like extensive grassland were actually comprised of very different habitat types. These heterogeneities and data gaps also weakened the robustness and power of the statistical analysis.
2. Other likely relevant data were not available at all, or not available quantitatively, so that potentially relevant information was lacking to complete the picture (e.g., organic fertilizer input, microstructures at the landscape level (e.g., field edges, hedgerows, habitat connectivity, pasture vs. stable feeding of cattle) or effects of increasing or decreasing commodity prices). In other cases, e.g., when analyzing the indirect effects of herbicides, potential correlations could not be studied due to a lack of quantifiability of potentially relevant parameters.
3. In our approach, we compared data of very high resolutions (insect biomass data of Hallmann et al. [14] and Mühlethaler et al. [42] which were generated at specific sites) with low resolution data (all other datasets at the federal state level). This is constrained by the lack of availability of truly local accurate long-term datasets for most of the relevant parameters. We postulate that overall, the federal state level data are sufficiently representative for the local conditions to establish meaningful correlations, being nevertheless aware that this representativeness may be limited in individual cases.
4. Furthermore, it is an important limitation of the approach that a retrospective analysis of factors and parameters can reveal correlations, but inherently is not capable of implying or proving causations. This can be an issue in particular in cases where multiple drivers are changing simultaneously. However, our analysis can suggest specific factors to be further examined as causal factors for insect decline through correlations. The authors should discuss the results and how they can be interpreted from the perspective of previous studies and of the working hypotheses. The findings and their implications should be discussed in the broadest context possible. Future research directions may also be highlighted.
5. Finally, a key limitation of the analysis lies in the granularity of the factor levels used. Many factors are interconnected through causal relationships, meaning some may exhibit significant correlations only within the context of a broader causal network. For instance, urbanization encompasses various elements, such as streets and gardens. While streets alone may not correlate strongly with insect decline, their interaction with other urbanization components, like housing, can strengthen the overall correlation. Conversely, gardens may mitigate the impact of urbanization by serving as biodiversity refuges, thus acting as confounding variables within the urbanization factor. The analysis accounts for the smallest possible facets of each factor, con-strained by data availability and correlation strength. Although urbanization shows a clear correlation with insect decline, further granularity is limited by the available data. While broader factor levels inherently include more confounding variables, this limitation does not weaken the validity of the analysis at the chosen level.

## 5. Conclusions

The decline of insect populations is a multifactorial phenomenon influenced by various factors acting over long time periods, mainly on a landscape level, and entailing changes in landscape management and landscape structures. In addition, many of these factors are interwoven with each other in complex ways

During the study period, urban and forested areas increased, whereas agricultural land decreased; though these were significant (especially urban areas, +20%), land use change overall was not dramatic (agricultural land −6%). Arable and grassland management shifted towards higher productivity. In agricultural areas, silage and bioenergy crops strongly increased, whereas permanent grassland, pastures, and fallow land decreased. Grassland was especially subject to major changes in management, with overall management intensity as well as areas of intensively managed grassland increasing.

Among the main drivers behind this development included: (a) a significant intensification in livestock and dairy farming practices, which led on the one hand to an increased demand of feed, and on the other hand to an increasing production of liquid manure, which is partly used as organic fertilizer, e.g., on grassland, and (b) the subsidization of bioenergy plants. Together, these factors led to a structural depletion of grassland and the conversion of grassland into bioenergy and silage crops.

The decline in the number of agricultural operations has led to a drastic increase in cultivated areas per farm, reflecting the need for higher management intensity and productivity to maintain the viability of an operation. Though there are no specific quantitative data, it can be assumed that, along with the decrease in farm numbers and increase in farm sizes, field sizes likewise increased, and the structural diversity of the agricultural landscape decreased.

In the meantime, the overall toxic load of pesticide exposure of arthropods was variable between the years, but decreasing by trend, which can likely be attributed to tightening environmental safety regulations. Climate and weather factors did not show obvious patterns that would concur with FIB decline, but they are likely to affect or act as stressors when functioning as confounding factors.

Overall, these observations make the hypothesis that the factors that most obviously concur with FIB decline are acting on a landscape level and entail changes in landscape management and structures plausible.

Land use change primarily took place in terms of urban areas and a decrease in permanent grassland, especially pastures; however, there was no major conversion of natural habitats into agricultural land, and overall agricultural areas decreased (Figure 4).

However, there is a broad spectrum of aspects of land use intensification, i.e., management intensification within a given land use type, the development of which coincides with FIB decline, and which are plausible as potential drivers. Most of them influence the availability of insect habitats or relevant structures therein, and therewith have the potential to affect insect abundance as well as diversity.

Overall, it can thus be concluded that intensification of land use and landscape management are likely to be key drivers of insect decline, which is confirming and reinforcing some of the key conclusions of IPBES 2018 [207] and FAO 2019 [208]. Meanwhile, as described above, land use comprises diverse components with varying characteristics, some of which show clear correlations and causal relationships with insect decline, while others act as confounding factors. Most factors do not operate independently but are embedded in complex networks of interdependencies. Consequently, significant correlations often emerge at the level of interconnected groups of factors or particularly strong standalone factors. One such interconnected group is agriculture, where agricultural intensification must be viewed as an overall package. Many facets of this intensification are intertwined and influence each other in a complex manner, further contributing to the interconnectedness of these factors.

The results of our study suggest that improved landscape management, including an optimized balance between extensively and intensively managed agricultural areas and natural habitats, as well as better conservation of structural diversity of the landscape, will be important approaches to counteract insect decline and protect insect biodiversity in central European landscapes.

## Figures and Tables

**Table 1:** The estimated coefficients, standard errors, t-values, and *p*-values for the linear regression model. The model was fitted to the reduced variable dataset, with insect biomass as dependent, and principal components 1 to 5 as independent variables. Highly significant effects are indicated by “***” for *p*-values less than 0.01 and trends by “.” for *p*-values less than 0.1.

Variable	Estimate	Std. Error	t	*p*	
PC1	0.42	0.07	5.9	<0.001	***
PC2	−0.11	0.18	−0.6	0.56	
PC3	−0.41	0.21	−1.9	0.07	.
PC4	0.18	0.24	0.8	0.46	
PC5	−0.17	0.26	−0.7	0.51	

## Data Availability

All data used in this article are presented in the Supplementary Materials. Data on pesticide applications broken down to the level of the individual data point can not be shared to comply with the use terms and conditions for the purchased database as stipulated by the supplier.

## Supplementary Materials

The following supporting information can be downloaded at: www.mdpi.com/article/10.3390/insects15121021/s1, Supplementary File S1—Data of all variables analyzed; Supplementary File S2—Regulatory endpoints used for toxic load calculations of pesticides; Supplementary File S3—Example of toxic load calculation; Supplementary File S4—Insecticidal seed treatments in Germany; Supplementary File S5—Additional figures: Figure S1: Normalization of the biomass data of Hallmann et al. (2017); Figure S2: Overview of variables with imputed datapoints; Figure S3: Heatmaps of various categories of variables; Figure S4: Additional figures of applied pesticides; Supplementary File S6—Statistical analysis: Additional methods and result section; Figure S1: Cosine square values (cos2) for all variables in the reduced variable dataset for principal components 1–5; Figure S2. Biplot with visual representation of the relationships between variables for the full variable data set in the PCA; Figure S3 Cosine square values (cos2) for all variables in the full variable dataset for principal components 1–4; Table S1. The estimated coefficients, standard errors, t-values, and *p*-values for the linear regression model.
