# Supplementary material for: Insect Decline—Evaluation of Potential Drivers of a Complex Phenomenon
Source: Insects. 2024 Dec 23;15(12):1021. doi: 10.3390/insects15121021 (PMC11676483; doi:10.3390/insects15121021)
Supplement: Supplementary file 1 [file insects-15-01021-s001.zip › insects-3278199-supplementary/Appendix E - Figures.pdf]

Insect decline –Evaluation of potential drivers of a complex phenomenon

Michael E. Grevé, Michael Thomas Marx, Sascha Eilmus, Matthias Ernst, John D. Herrmann, Christian Ulrich Baden, Christian Maus

Appendix E

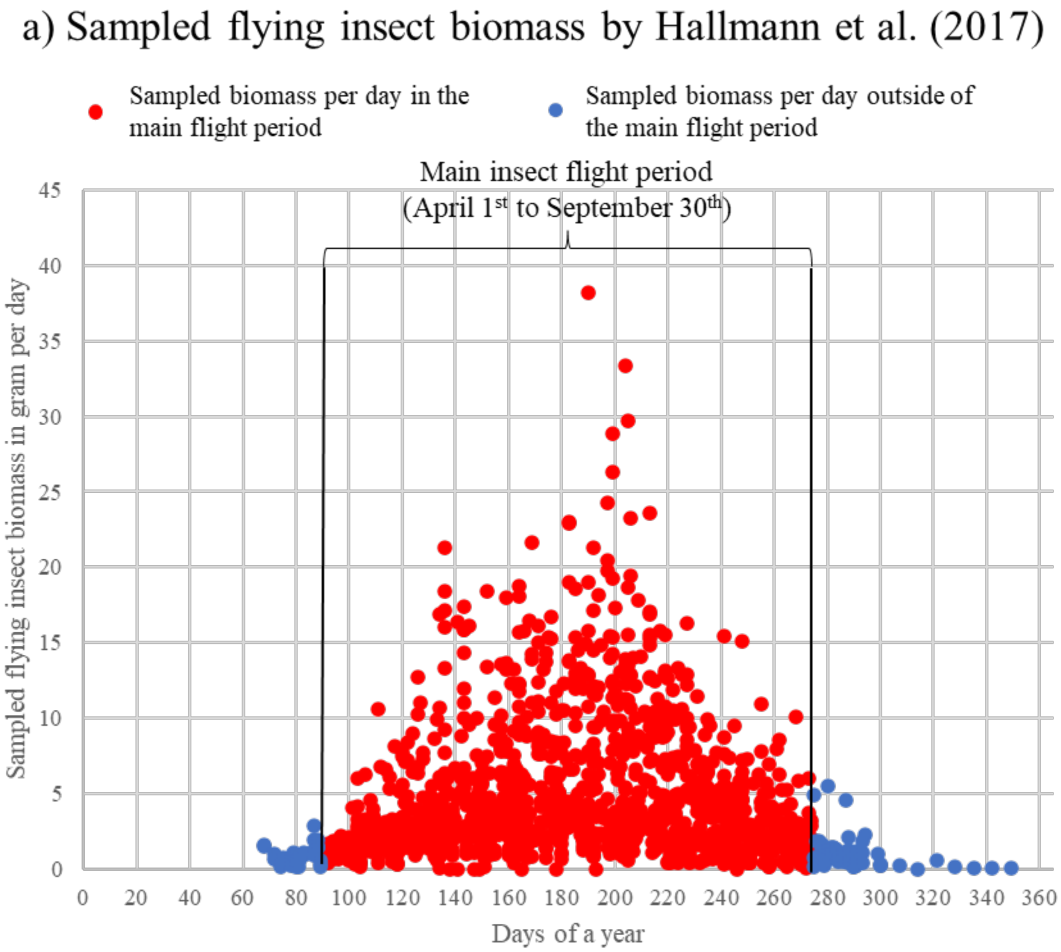

a) Sampled flying insect biomass by Hallmann et al. (2017)
Sampled biomass per day in the main flight period
Sampled biomass per day outside of the main flight period
Main insect flight period
(April 1st to September 30th)
Sampled flying insect biomass in gram per day
Days of a year
0
5
10
15
20
25
30
35
40
45
0 20 40 60 80 100 120 140 160 180 200 220 240 260 280 300 320 340 360

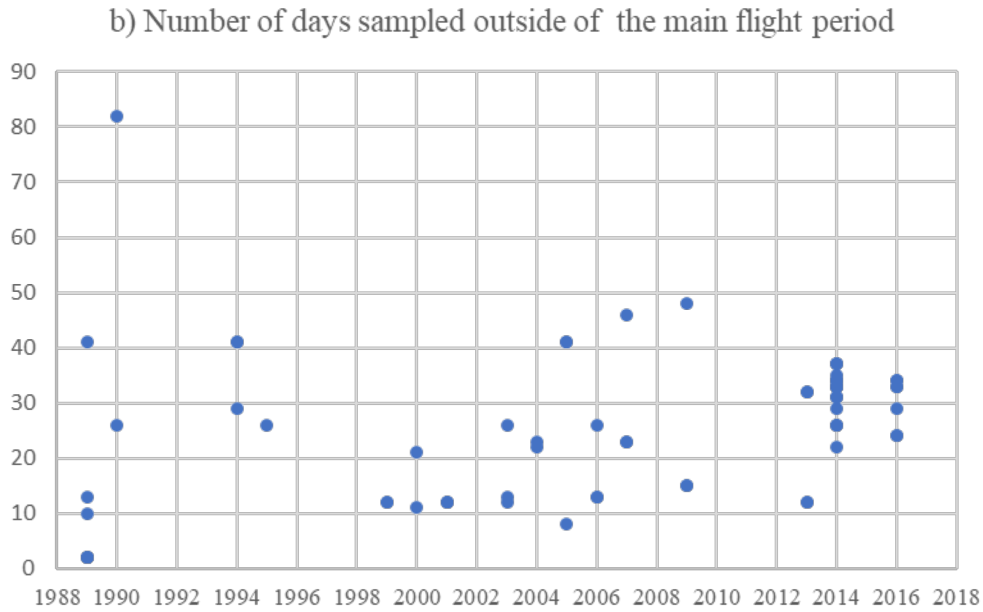

b) Number of days sampled outside of the main flight period
0
10
20
30
40
50
60
70
80
90
1988 1990 1992 1994 1996 1998 2000 2002 2004 2006 2008 2010 2012 2014 2016 2018

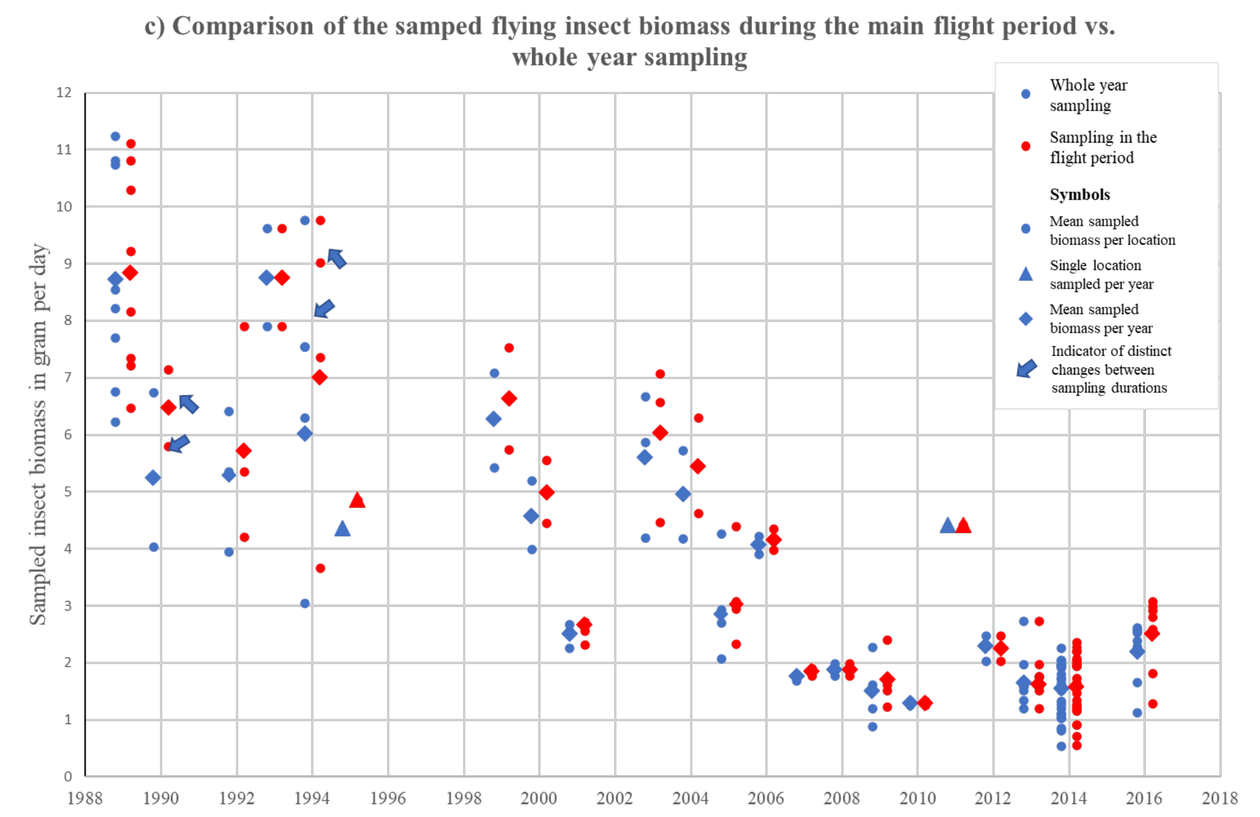

Figure S1: Normalization of the biomass data set of the sampled flying insect biomass of the study by Hallmann et al. (2017). The Supplementary Material of Hallmann et al. (2017) includes detailed information on the biomass of insects sampled over a defined period of days per sampling location. For this study, we focused on the main flight period of insects, which roughly spans from April 1st to September 30th each year.

Figure S1 a) Flying insect biomass data caught by Hallmann et al. (2017) within and outside of the main flight period. Each datapoint represents the sampled flying insect biomass in gram per day of each sampling period which is the number of days that a Malaise trap is collecting insects before they are emptied; b) The number of days where flying insect biomass was collected outside of the main insect flight period over the years of the study by Hallmann et al. (2017); c) Comparison of the sampled flying insect biomass data collected over the whole year (blue) and the main insect flight period (red). Each data point represents the average insect biomass sampled per location. Also, the annual mean values and years with only a single sampling location are shown.

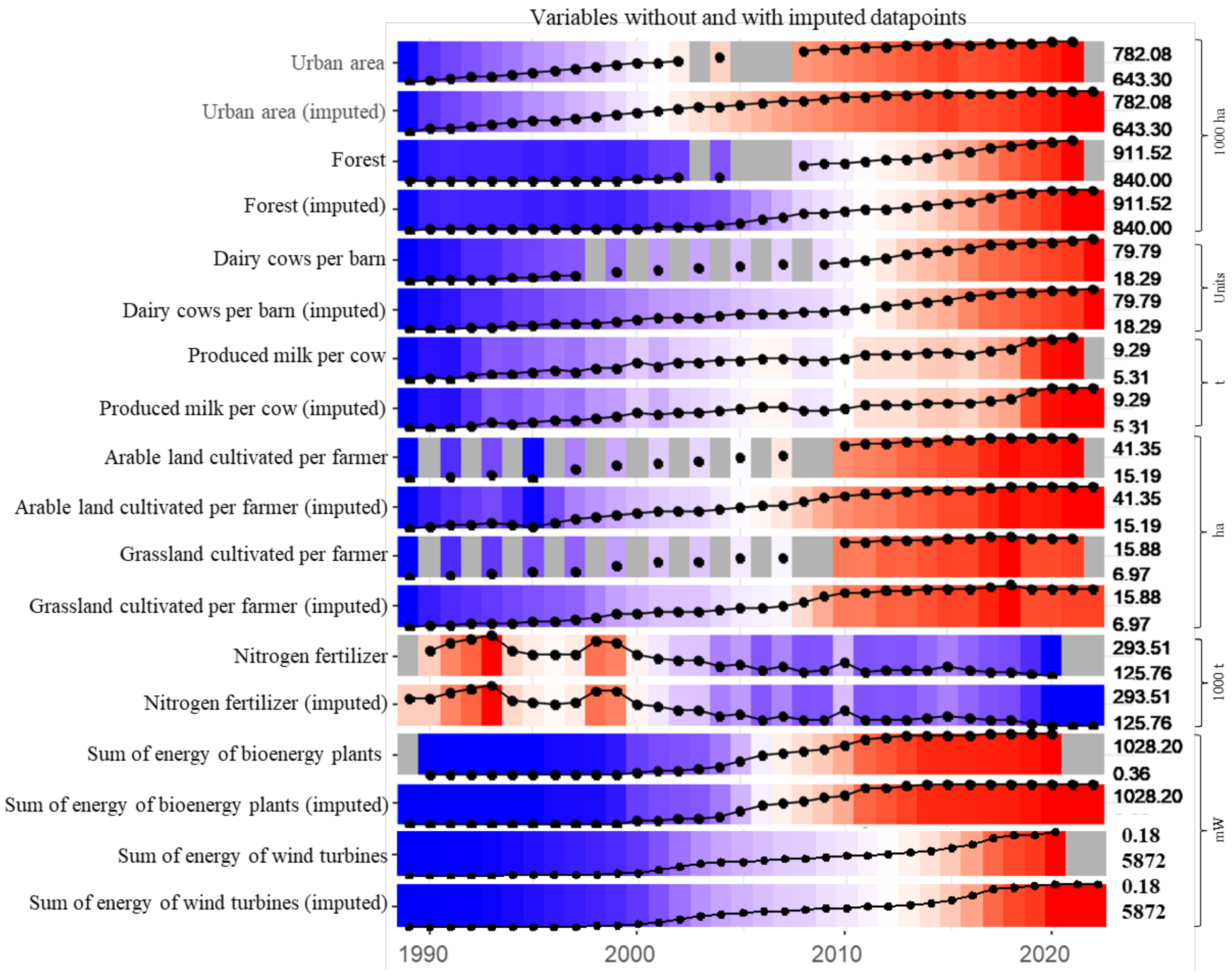

Figure S2: Overview of variables with imputed datapoints. Overview of the predictor variables that contained missing values and the same variables after the missing values using a linear imputation were added. The comparison of the original and the imputed variables is also shown in Appendix A, Sheet 4. The variables with imputed datapoints are used in the conducted random forest analysis.

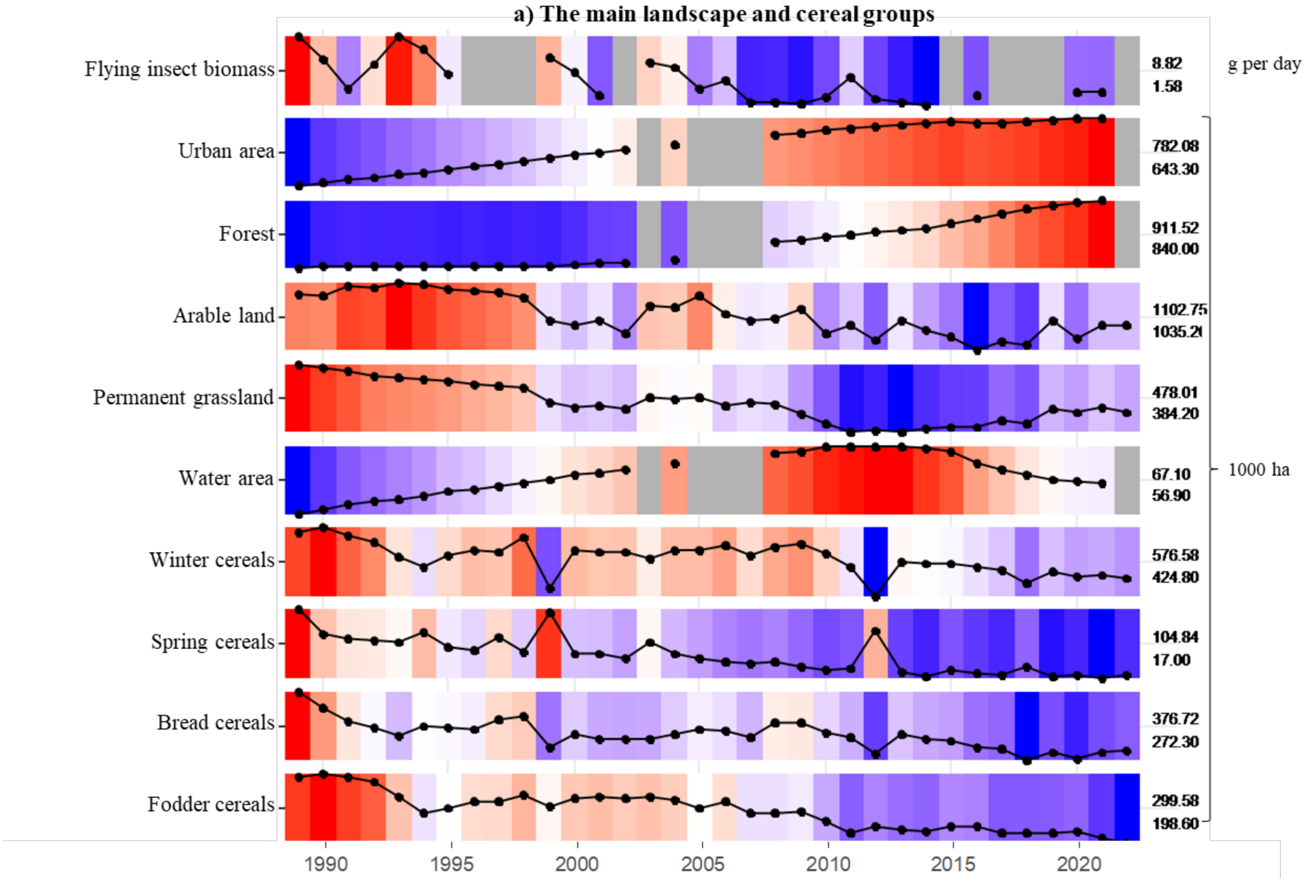

a) The main landscape and cereal groups
Flying insect biomass
Urban area
Forest
Arable land
Permanent grassland
Water area
Winter cereals
Spring cereals
Bread cereals
Fodder cereals
8.82
1.58
782.08
643.30
911.52
840.00
1102.75
478.01
384.20
67.10
56.90
576.58
424.80
104.84
17.00
376.72
272.30
299.58
198.60
g per day
1000 ha
1990
1995
2000
2005
2010
2015
2020

•

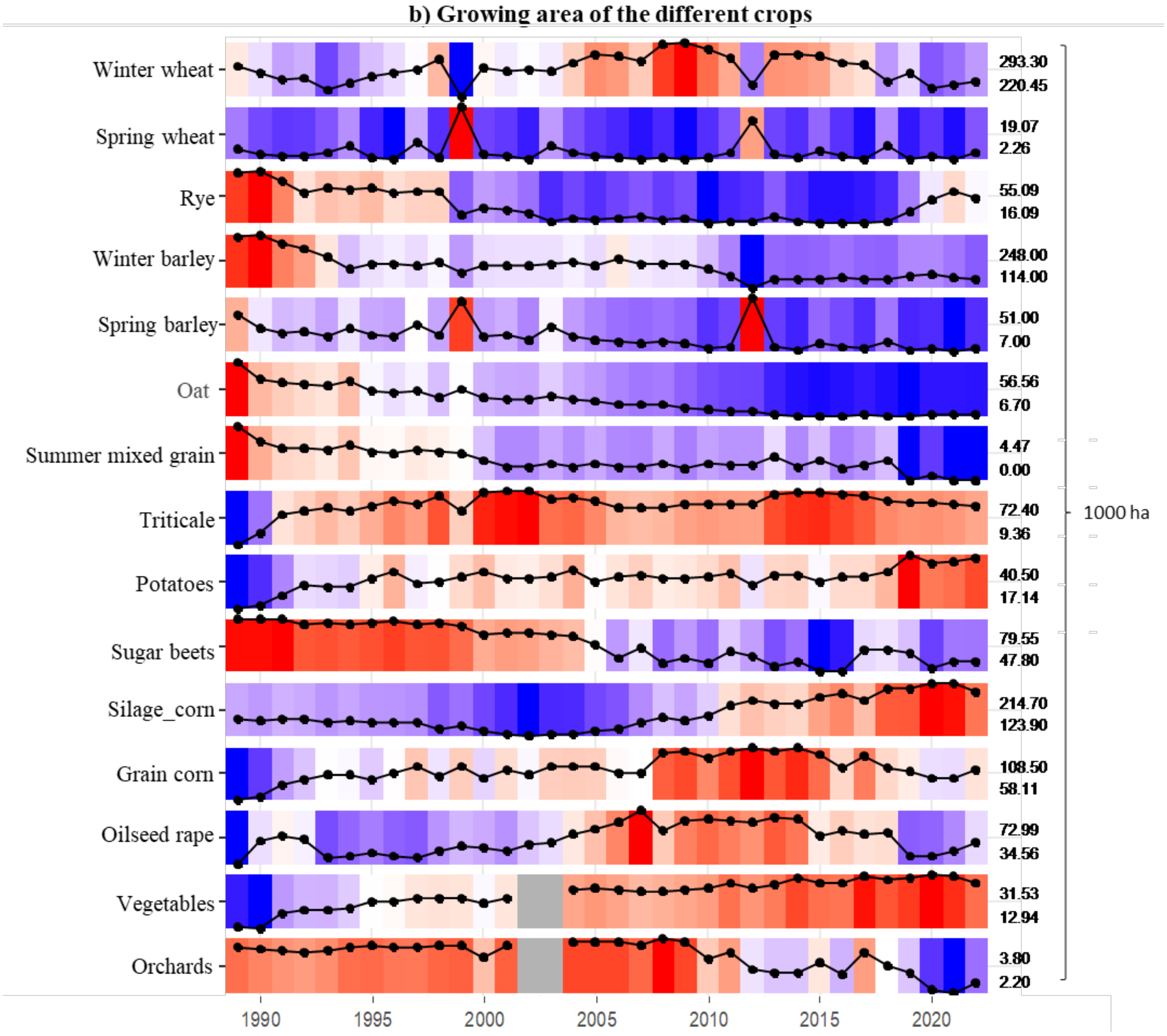

b) Growing area of the different crops
Winter wheat
Spring wheat
Rye
Winter barley
Spring barley
Oat
Summer mixed grain
Triticale
Potatoes
Sugar beets
Silage_corn
Grain corn
Oilseed rape
Vegetables
Orchards
293.30
220.45
19.07
2.26
55.09
16.09
248.00
114.00
51.00
7.00
56.56
6.70
4.47
0.00
72.40
9.36
40.50
17.14
79.55
47.80
214.70
123.90
108.50
58.11
72.99
34.56
31.53
12.94
3.80
2.20
1000 ha
1990
1995
2000
2005
2010
2015
2020

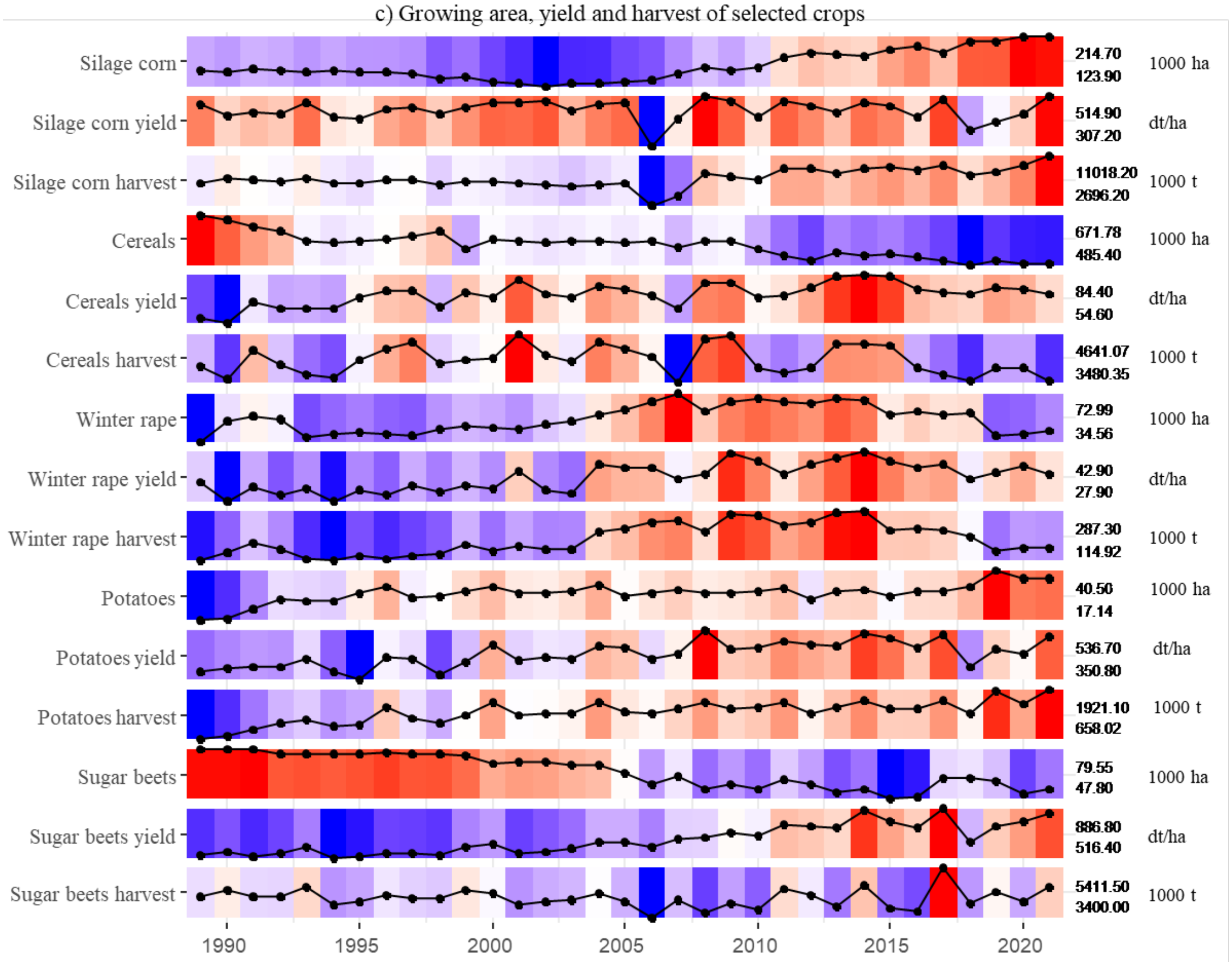
c) Growing area, yield and harvest of selected crops
Silage corn
214.70
123.90
1000 ha
Silage corn yield
514.90
307.20
dt/ha
Silage corn harvest
11018.20
2696.20
1000 t
Cereals
671.78
485.40
1000 ha
Cereals yield
84.40
54.60
dt/ha
Cereals harvest
4641.07
3480.35
1000 t
Winter rape
72.99
34.56
1000 ha
Winter rape yield
42.90
27.90
dt/ha
Winter rape harvest
287.30
114.92
1000 t
Potatoes
40.50
17.14
1000 ha
Potatoes yield
536.70
350.80
dt/ha
Potatoes harvest
1921.10
658.02
1000 t
Sugar beets
79.55
47.80
1000 ha
Sugar beets yield
886.80
516.40
dt/ha
Sugar beets harvest
5411.50
3400.00
1000 t
1990
1995
2000
2005
2010
2015
2020

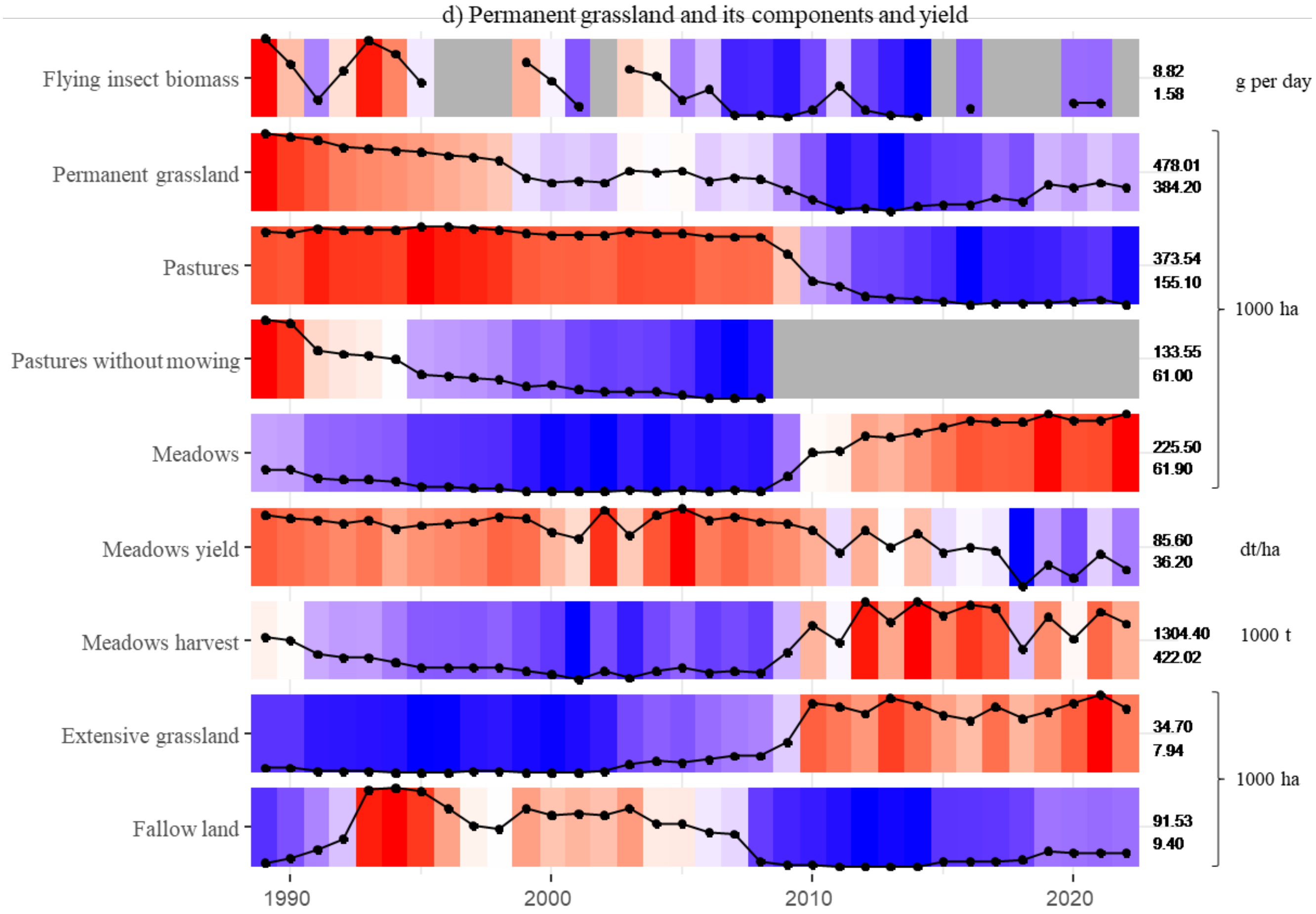
d) Permanent grassland and its components and yield
Flying insect biomass
Permanent grassland
Pastures
Pastures without mowing
Meadows
Meadows yield
Meadows harvest
Extensive grassland
Fallow land
8.82
1.58
g per day
478.01
384.20
373.54
155.10
133.55
61.00
225.50
61.90
1000 ha
85.60
36.20
dt/ha
1304.40
422.02
1000 t
34.70
7.94
91.53
9.40
1000 ha
1990
2000
2010
2020

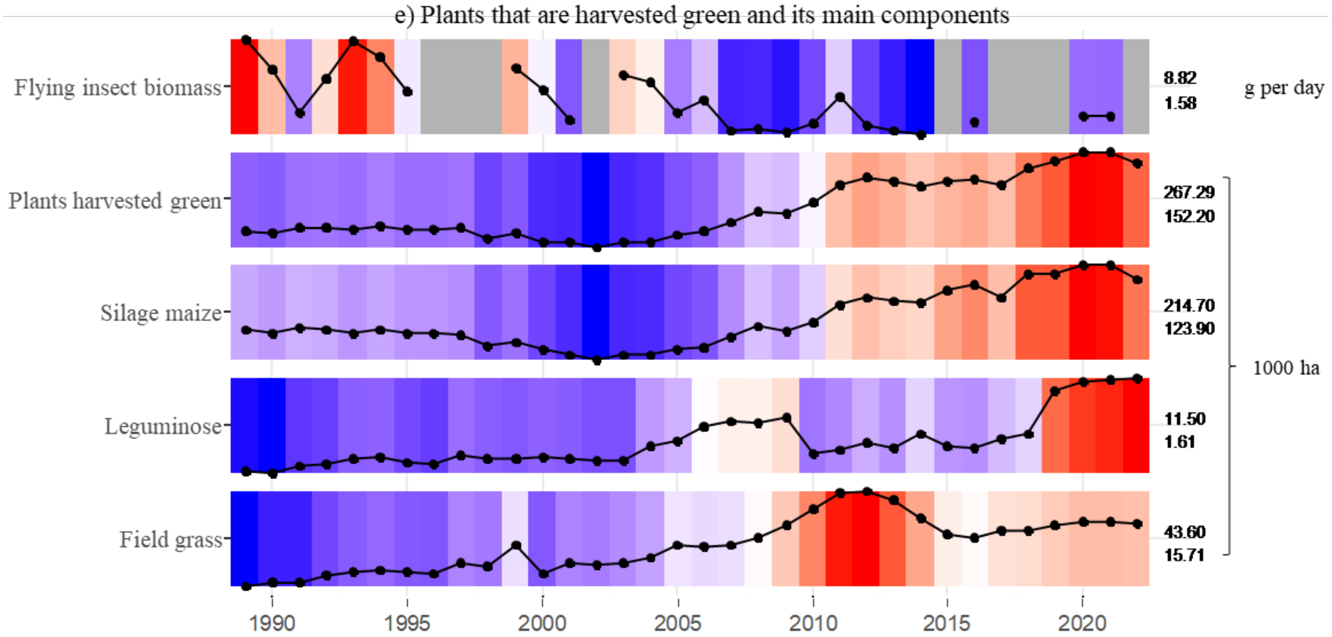
e) Plants that are harvested green and its main components
Flying insect biomass
Plants harvested green
Silage maize
Leguminose
Field grass
8.82
1.58
g per day
267.29
152.20
214.70
123.90
11.50
1.61
43.60
15.71
1000 ha
1990
1995
2000
2005
2010
2015
2020

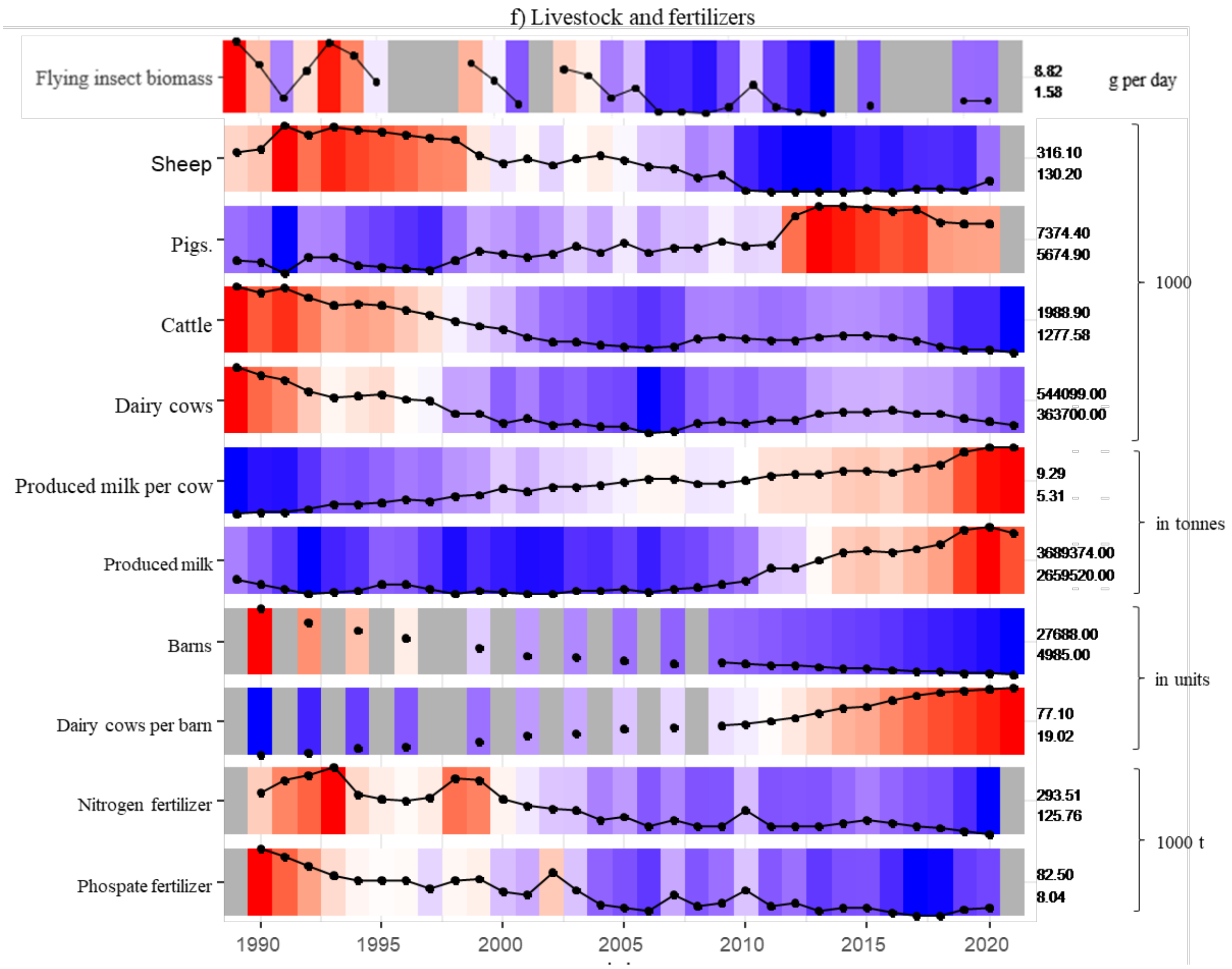

f) Livestock and fertilizers
Flying insect biomass
8.82
1.58
g per day
Sheep
316.10
130.20
Pigs.
7374.40
5674.90
1000
Cattle
1988.90
1277.58
Dairy cows
544099.00
363700.00
Produced milk per cow
9.29
5.31
in tonnes
Produced milk
3689374.00
2659520.00
Barns
27688.00
4985.00
in units
Dairy cows per barn
77.10
19.02
Nitrogen fertilizer
293.51
125.76
1000 t
Phospate fertilizer
82.50
8.04
1990
1995
2000
2005
2010
2015
2020

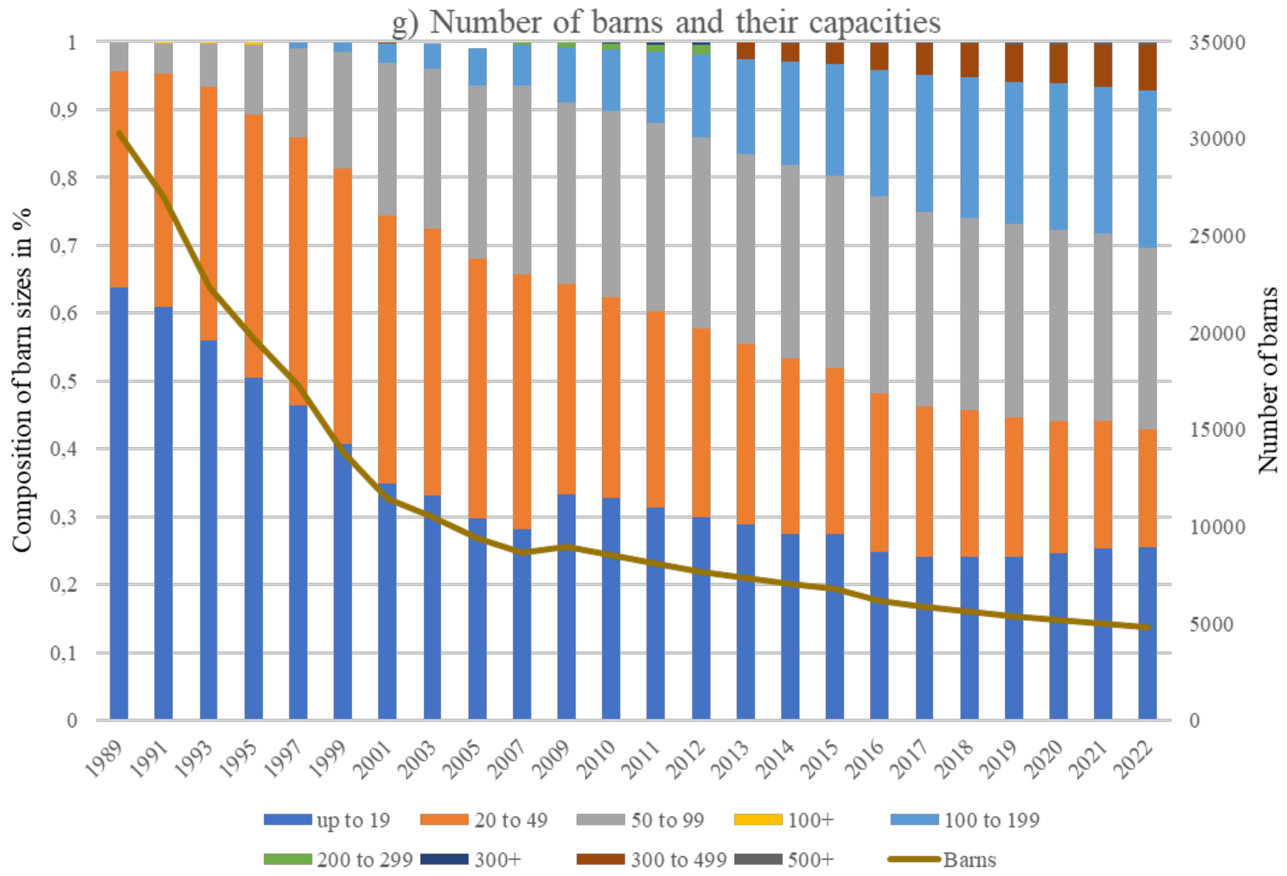
g) Number of barns and their capacities
Composition of barn sizes in %
Number of barns
1
0,9
0,8
0,7
0,6
0,5
0,4
0,3
0,2
0,1
0
35000
30000
25000
20000
15000
10000
5000
0
1989
1991
1993
1995
1997
1999
2001
2003
2005
2007
2009
2010
2011
2012
2013
2014
2015
2016
2017
2018
2019
2020
2021
2022
up to 19
20 to 49
50 to 99
100+
100 to 199
200 to 299
300+
300 to 499
500+
Barns

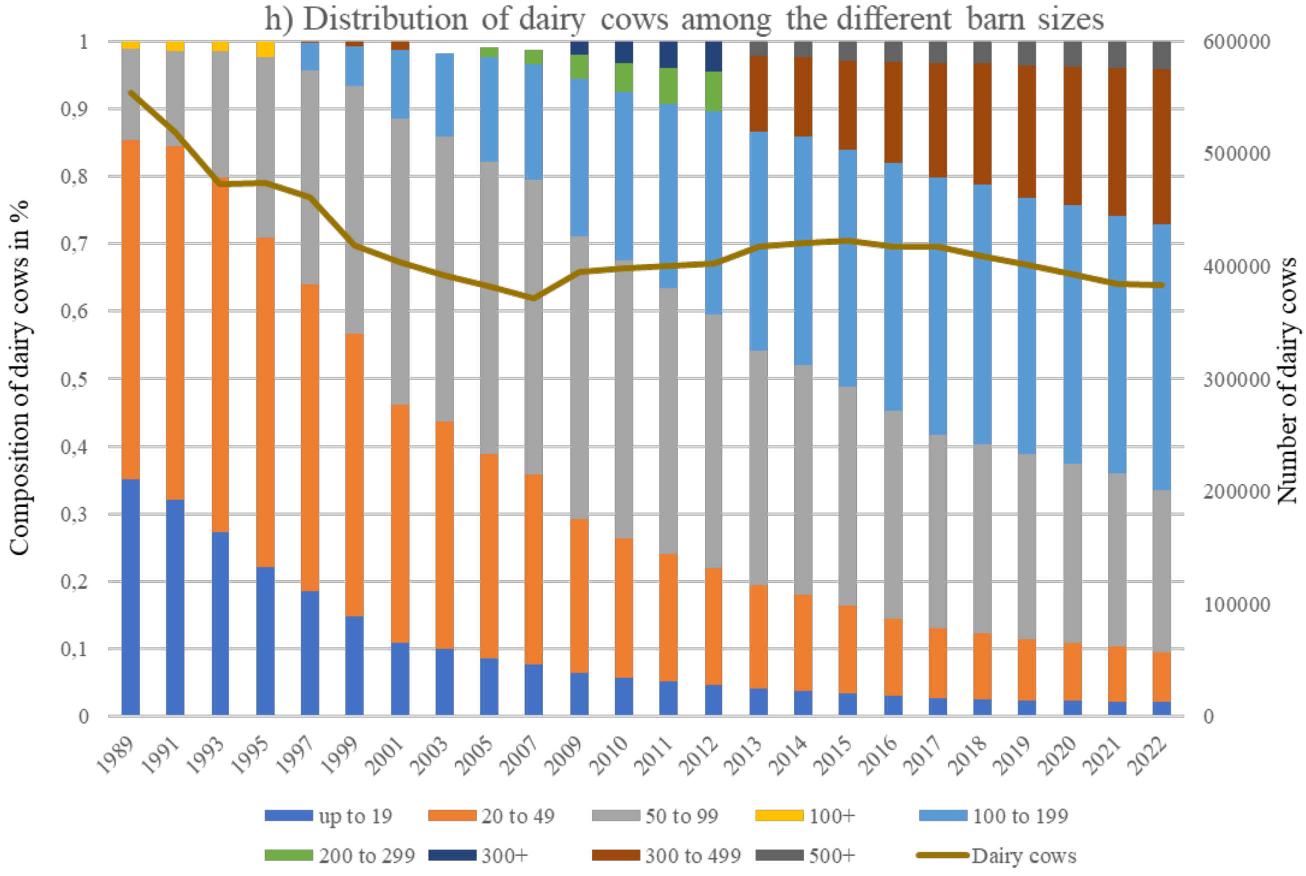
h) Distribution of dairy cows among the different barn sizes
Composition of dairy cows in %
Number of dairy cows
1
0,9
0,8
0,7
0,6
0,5
0,4
0,3
0,2
0,1
0
600000
500000
400000
300000
200000
100000
0
1989
1991
1993
1995
1997
1999
2001
2003
2005
2007
2009
2010
2011
2012
2013
2014
2015
2016
2017
2018
2019
2020
2021
2022
up to 19
20 to 49
50 to 99
100+
100 to 199
200 to 299
300+
300 to 499
500+
Dairy cows

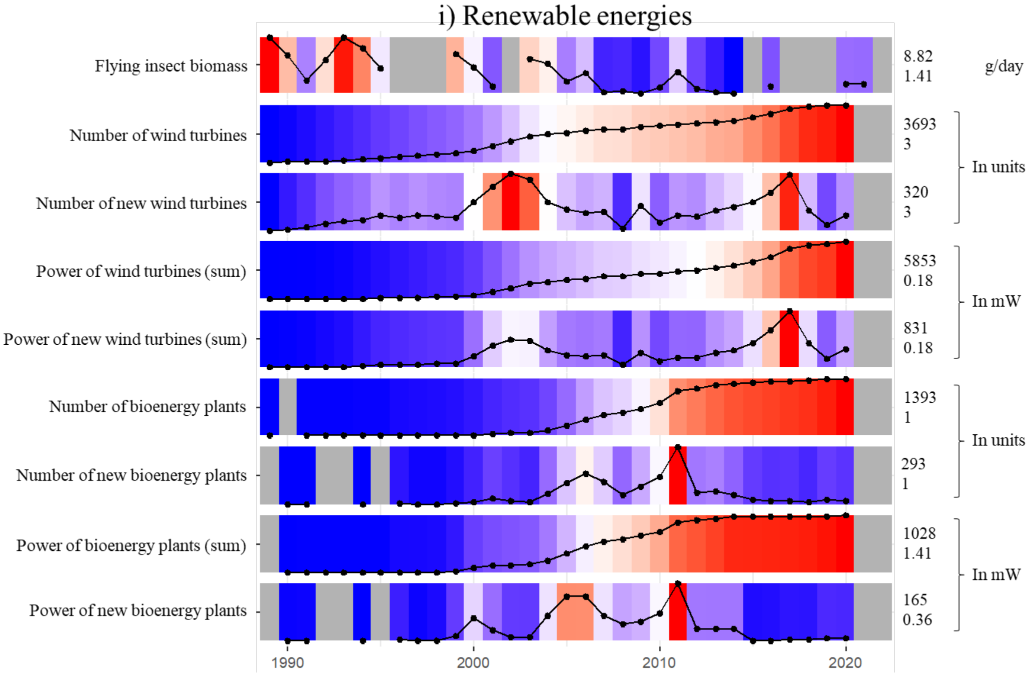

Figure S3: Heatmaps of various categories of variables.

The presentation includes the annual mean flying insect biomass, obtained from Hallmann et al.'s research in 2017. The figure is organized into various sections: a) The main landscape types and cereal classes; b) The growing areas of different crops; c) The growing area, yield, and harvest of selected crops; d) The primary components of permanent grassland; e) Plants that are harvested green and their main components; f) Livestock and fertilizers; g) The composition of dairy cow barn sizes and the number of barns with dairy cows; h) The distribution of dairy cows among different barn sizes; i) Renewable energies; To aid comparability, the annual mean flying insect biomass is also reiterated at the top of most figures for better analysis and understanding.

A

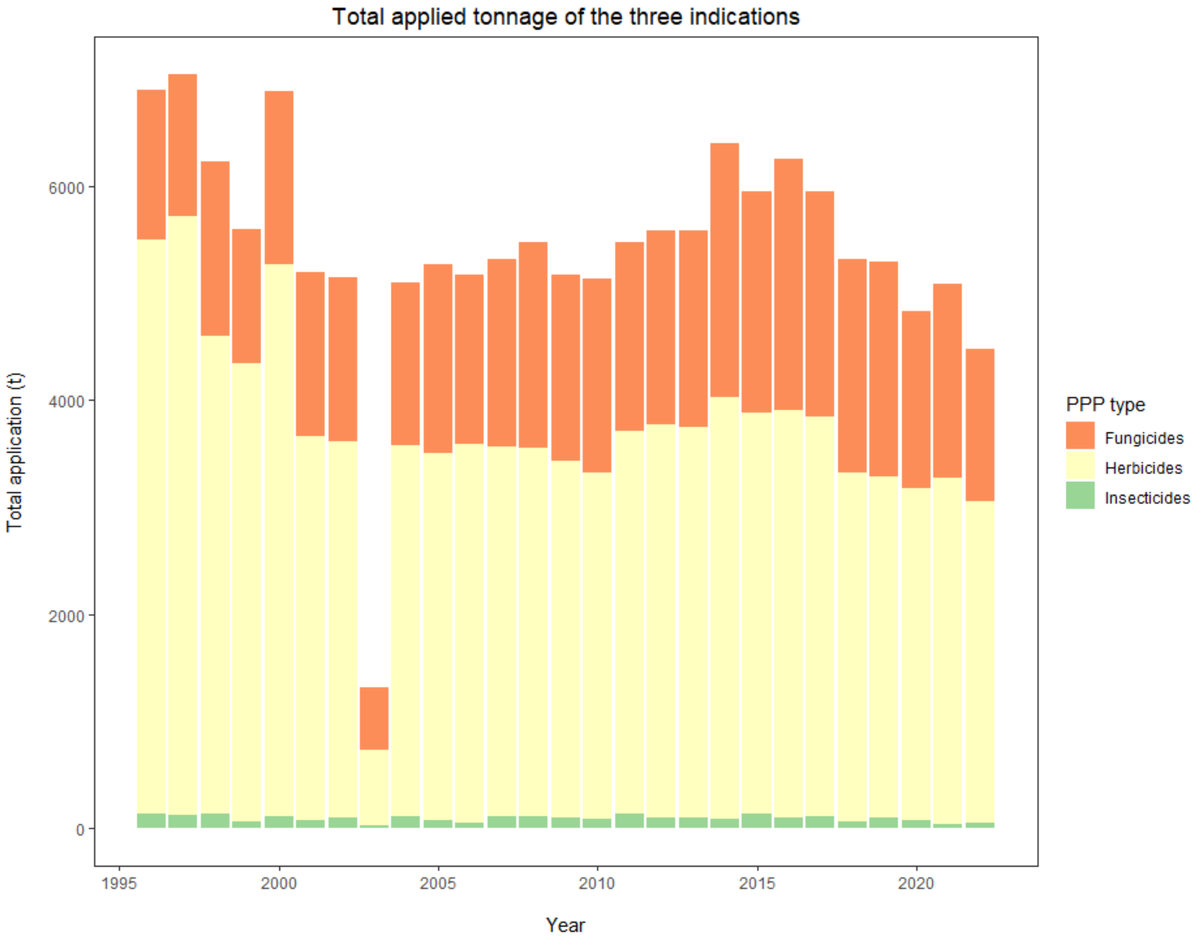

Total applied tonnage of the three indications
Total application (t)
6000
4000
2000
0
1995
2000
2005
2010
2015
2020
Year
PPP type
Fungicides
Herbicides
Insecticides

B Toxic load of insecticidal seed treatments per crop

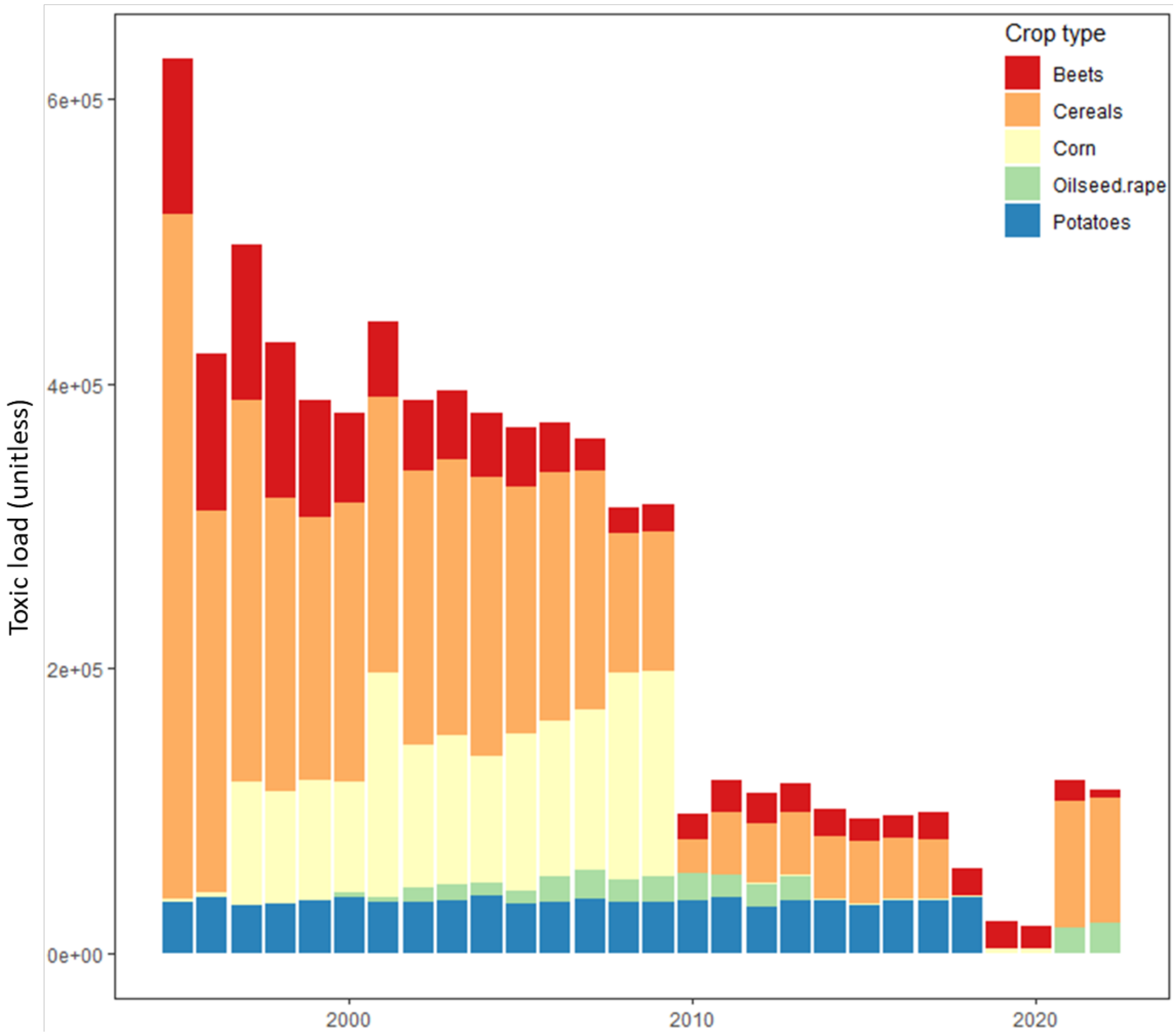

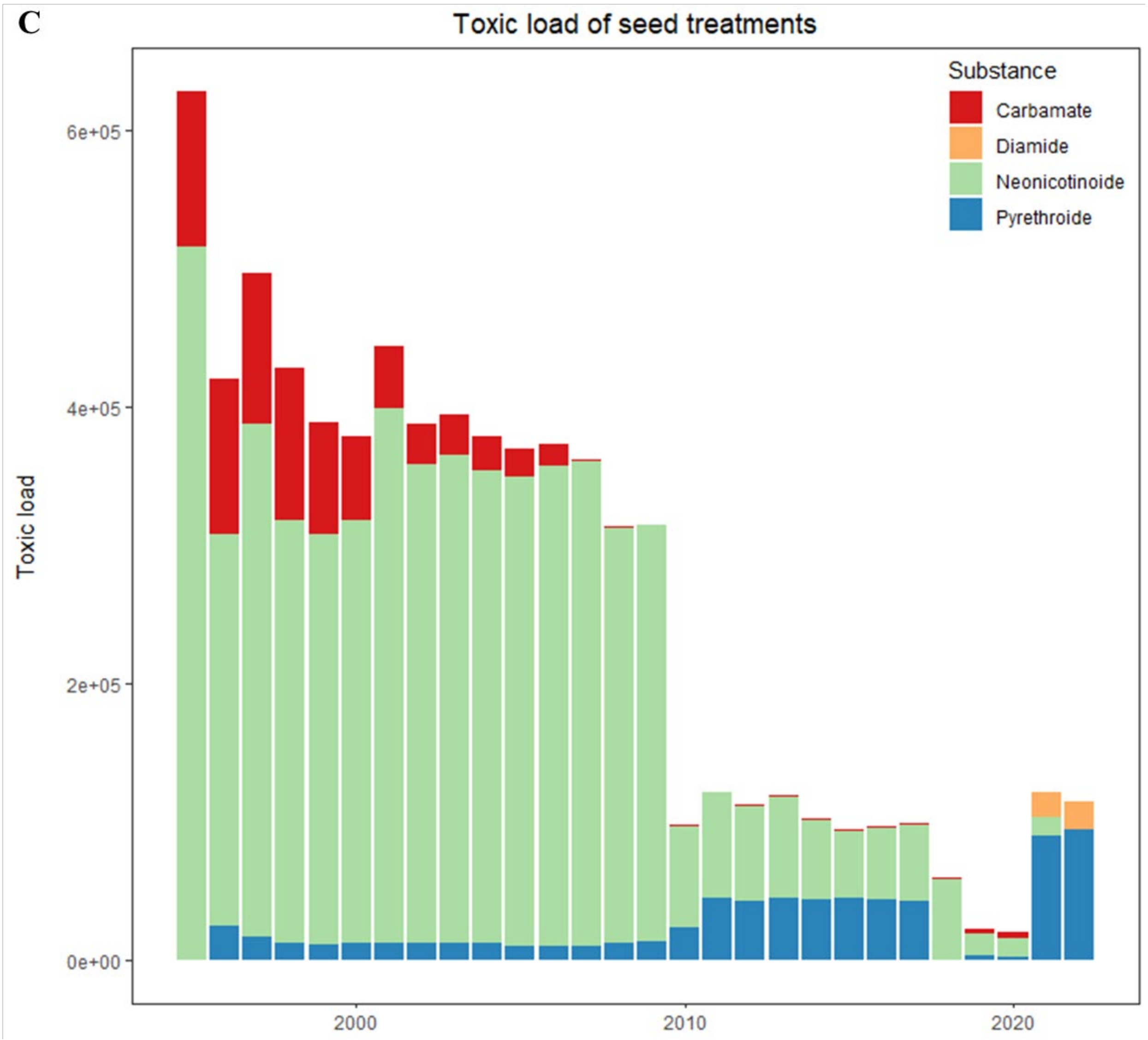
C
Toxic load of seed treatments
Substance
Carbamate
Diamide
Neonicotinoide
Pyrethroide
Toxic load
6e+05
4e+05
2e+05
0e+00
2000
2010
2020

D

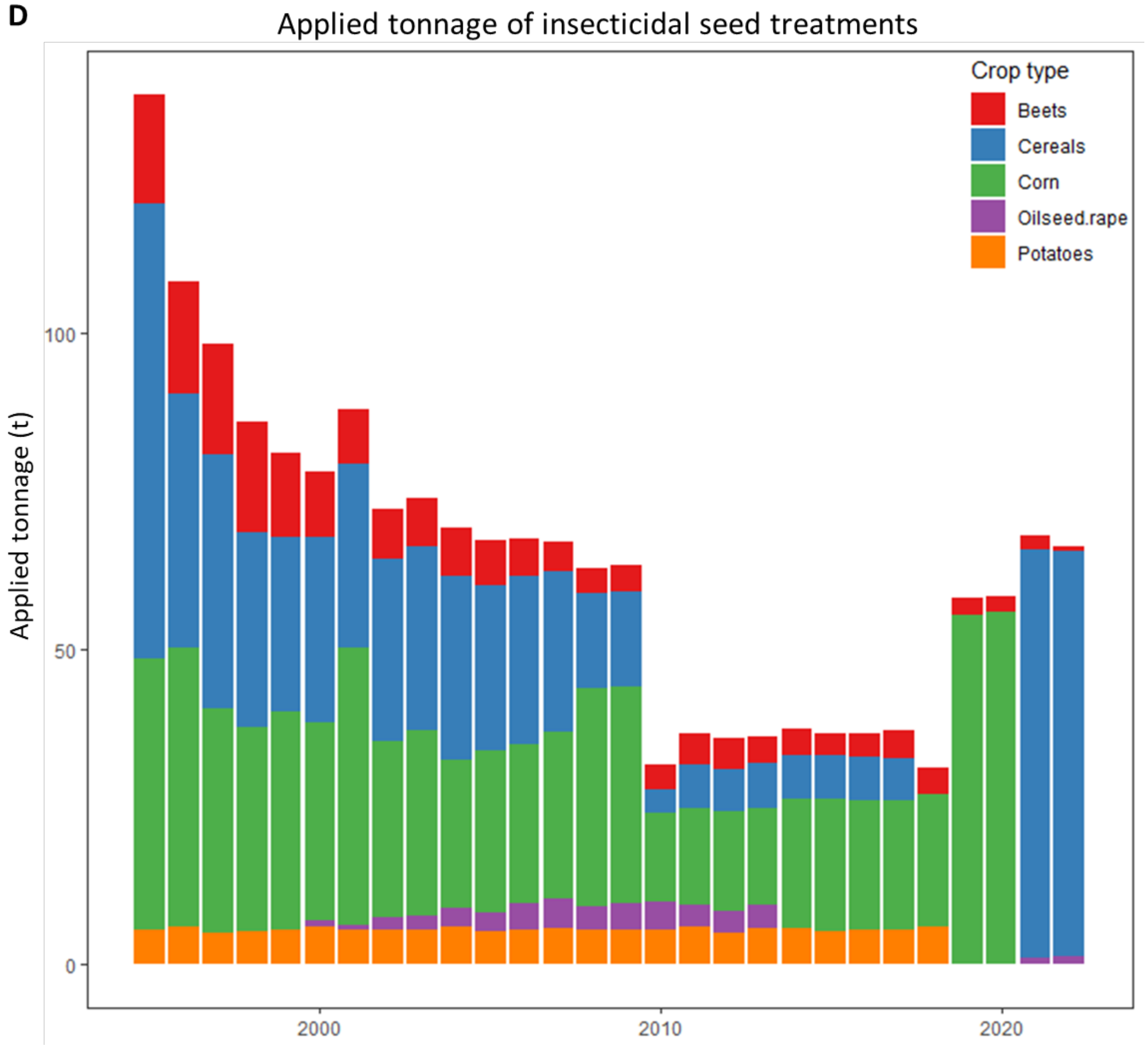
Applied tonnage of insecticidal seed treatments
Applied tonnage (t)
0
50
100
2000
2010
2020
Crop type
Beets
Cereals
Corn
Oilseed.rape
Potatoes

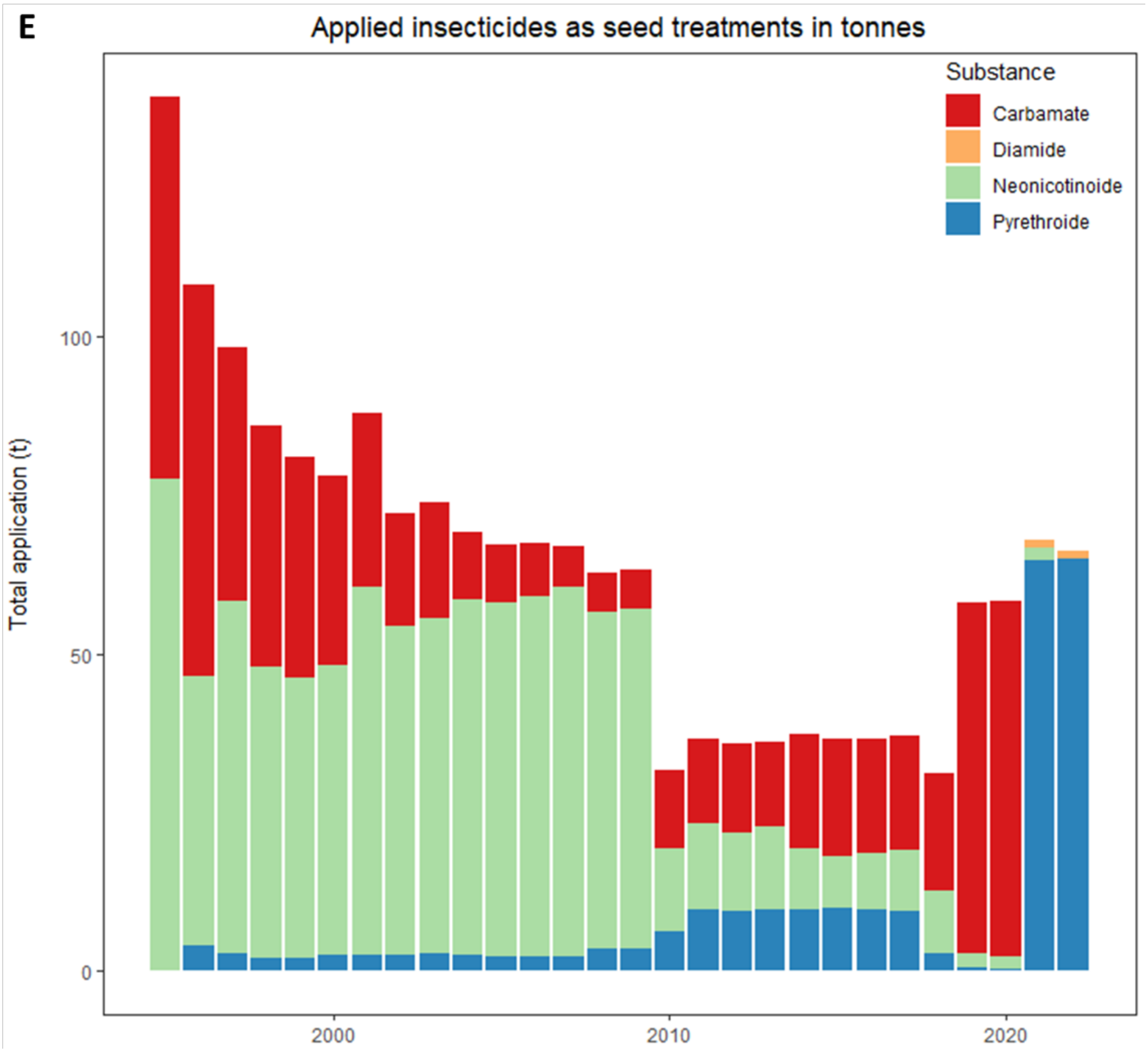

Figure S4: Additional figures of applied pesticides.

A) applied tonnage of the three indications of foliar-applied pesticides, based on market research data, B) toxic load of insecticidal seed treatments per crop, and C) per indication and D) applied tonnage of insecticidal seed treatments per crop, and E) per indication.
