# Supplementary material for: Insect Decline—Evaluation of Potential Drivers of a Complex Phenomenon
Source: Insects. 2024 Dec 23;15(12):1021. doi: 10.3390/insects15121021 (PMC11676483; doi:10.3390/insects15121021)
Supplement: Supplementary file 1 [file insects-15-01021-s001.zip › insects-3278199-supplementary/Appendix F - Statistical analysis.pdf]

Insect decline –Evaluation of potential drivers of a complex phenomenon

Michael E. Grevé, Michael Thomas Marx, Sascha Eilmus, Matthias Ernst, John D. Herrmann, Christian Ulrich Baden, Christian Maus

Appendix F

Methods

Additional variable information

Overall, time series data of 92 variables (compare Appendix A and E) from multiple sources was gathered for this analysis of which 57 were related to agriculture, 8 to livestock farming, 3 to the broader landscape features such as "urban area", 8 to weather and climate, 10 to renewable energies, and 6 to pesticides. To condense the number of variables for the statistical analysis, 33 variables were selected for further analysis. All main landscape types such as urban area, arable land, meadows, or pastures were included when they covered at least 5% of NRW at any time of the study period.

Crops with similar types of cultivation, such as oat and winter wheat were summed up to the main cereal groups bread and fodder cereals. Other crops with different types of cultivations such as sugar beets were included if they covered 5% or more of the arable land at any time of the study period. We included the area of grassland and of arable land cultivated per farmer as well as the applied tonnage of nitrogen fertilizers. Use of fertilizers was illustrated by reference to nitrogen fertilizers; both nitrogen and phosphate fertilizers are artificial fertilizers, and both showed a declining trend over time (Appendix E, Fig F.), but nitrogen was applied at higher tonnages. For animal husbandry, we used the total numbers of the different livestock types, namely sheep, pigs, and cattle. For dairy farming we referred to the mean number of dairy cows per barn, as representative for animal housing, and moreover the produced milk per cow as they represent changes in the productivity of dairy farming. For renewable energies, we used the sum of generated power of bioenergy and wind turbines, and for pesticides we used the toxic load of all foliar pesticides, and the toxic load of the insecticidal seed treatments. In addition, all four analyzed climate/weather variables namely the average temperature over the whole year and the winter months previous to the samplings as well as the total precipitation over the whole year and the winter months.

Results

For each principial component, we additionally calculated the cosine square distribution for all predictors. Figure 1 A-E depict the cosine values for the PCA with the full biomass dataset and reduced variable set.

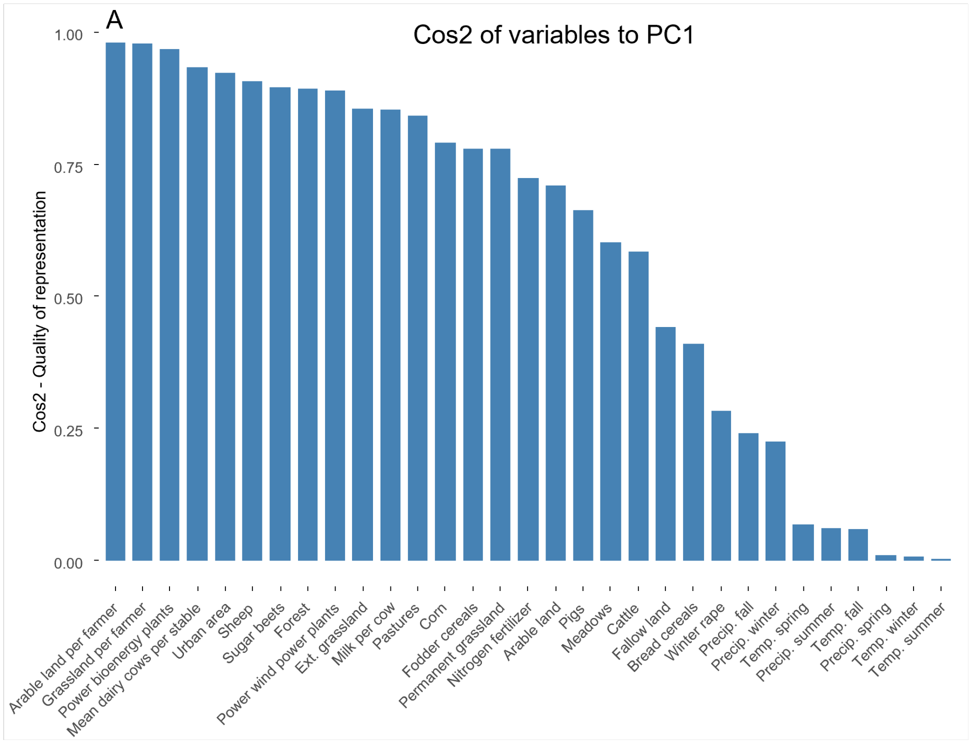

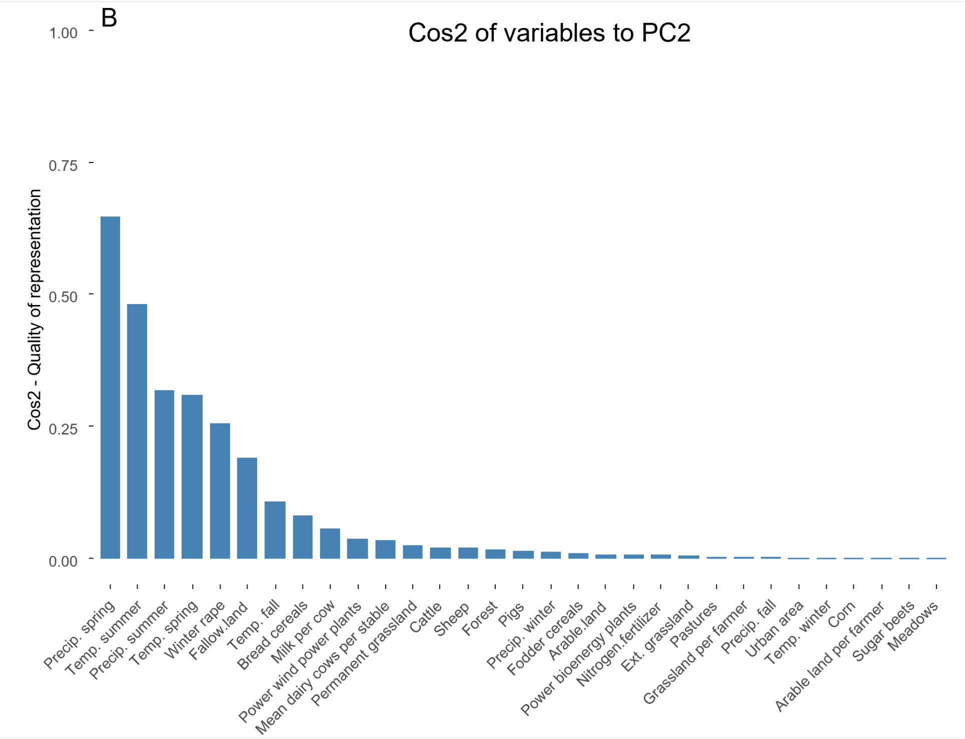
B
Cos2 of variables to PC2
Cos2 - Quality of representation
1.00
0.75
0.50
0.25
0.00
Precip. spring
Temp. summer
Precip. summer
Temp. spring
Winter rape
Fallow.land
Temp. fall
Bread cereals
Milk per cow
Power wind power plants
Mean dairy cows per stable
Permanent grassland
Cattle
Sheep
Forest
Pigs
Precip. winter
Fodder cereals
Arable.land
Power bioenergy plants
Nitrogen.fertilizer
Ext. grassland
Pastures
Grassland per farmer
Precip. fall
Urban area
Temp. winter
Corn
Arable land per farmer
Sugar beets
Meadows

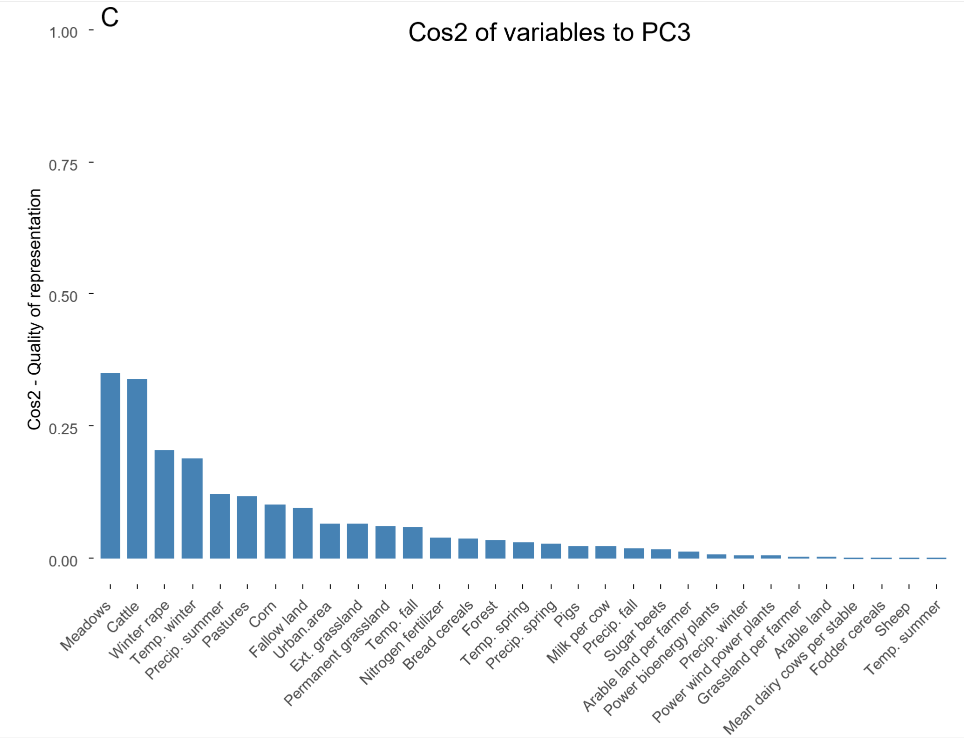
C
Cos2 of variables to PC3
Cos2 - Quality of representation
1.00
0.75
0.50
0.25
0.00
Meadows
Cattle
Winter rape
Temp. winter
Precip. summer
Pastures
Corn
Fallow land
Urban.area
Ext. grassland
Permanent grassland
Temp. fall
Nitrogen fertilizer
Bread cereals
Forest
Temp. spring
Precip. spring
Pigs
Milk per cow
Precip. fall
Sugar beets
Arable land per farmer
Power bioenergy plants
Precip. winter
Power wind power plants
Grassland per farmer
Arable land
Mean dairy cows per stable
Fodder cereals
Sheep
Temp. summer

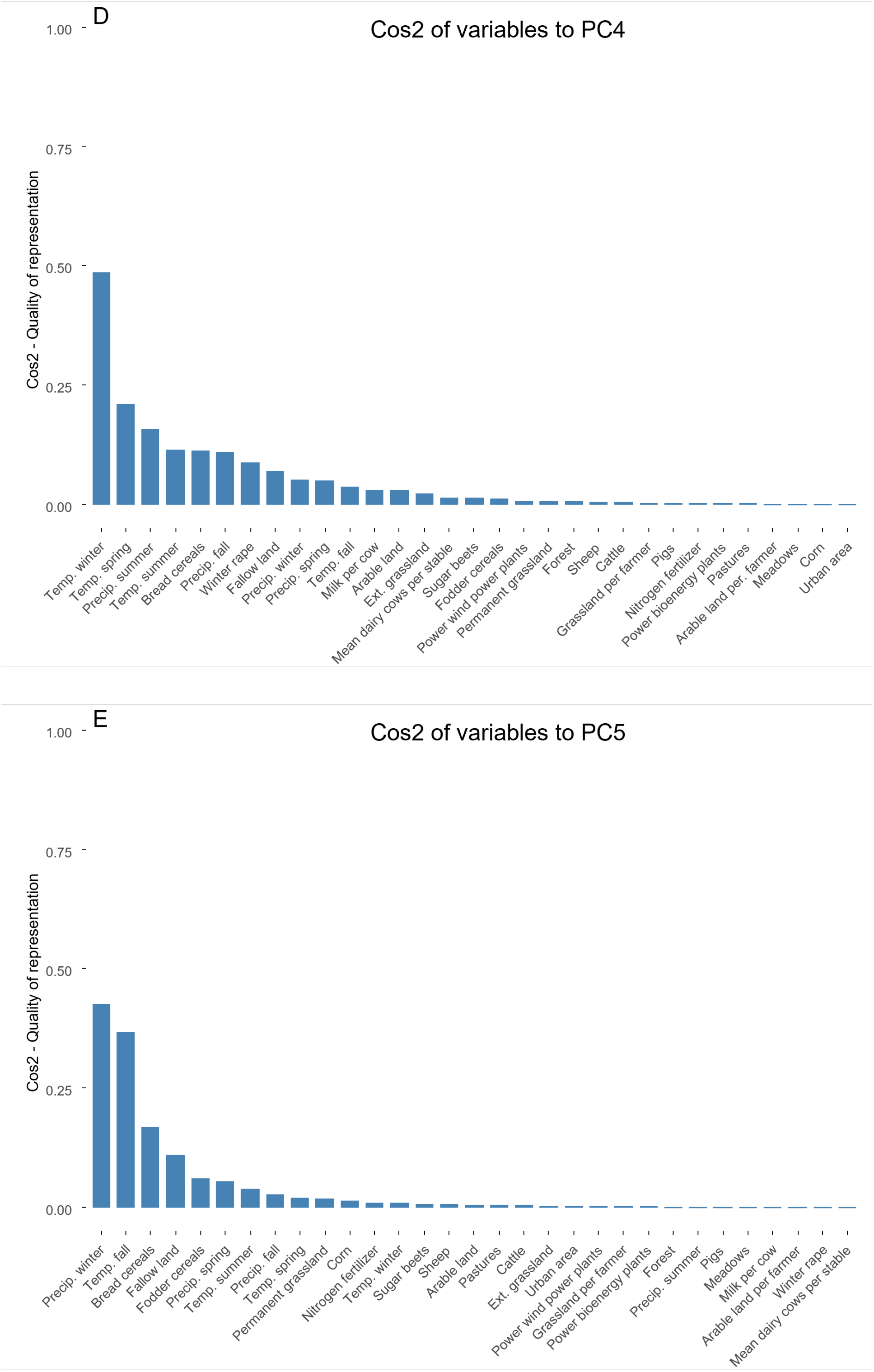

Figure S1 A-E depict cosine square values (cos2) for all variables in the reduced variable dataset for principal components 1 -5.

In the PCA for the sensitivity analysis with the reduced biomass dataset and full variable set, the PC1 is very similar to the PC1 calculated for the reduced variable dataset (Figure 3A) and can also broadly be described as an "agricultural intensification and land use change gradient." (Figure 2). However, the direction of the gradient is reversed in comparison to the biplot for the full biomass dataset and reduced variable set due to the random initialization of the PCA algorithm.

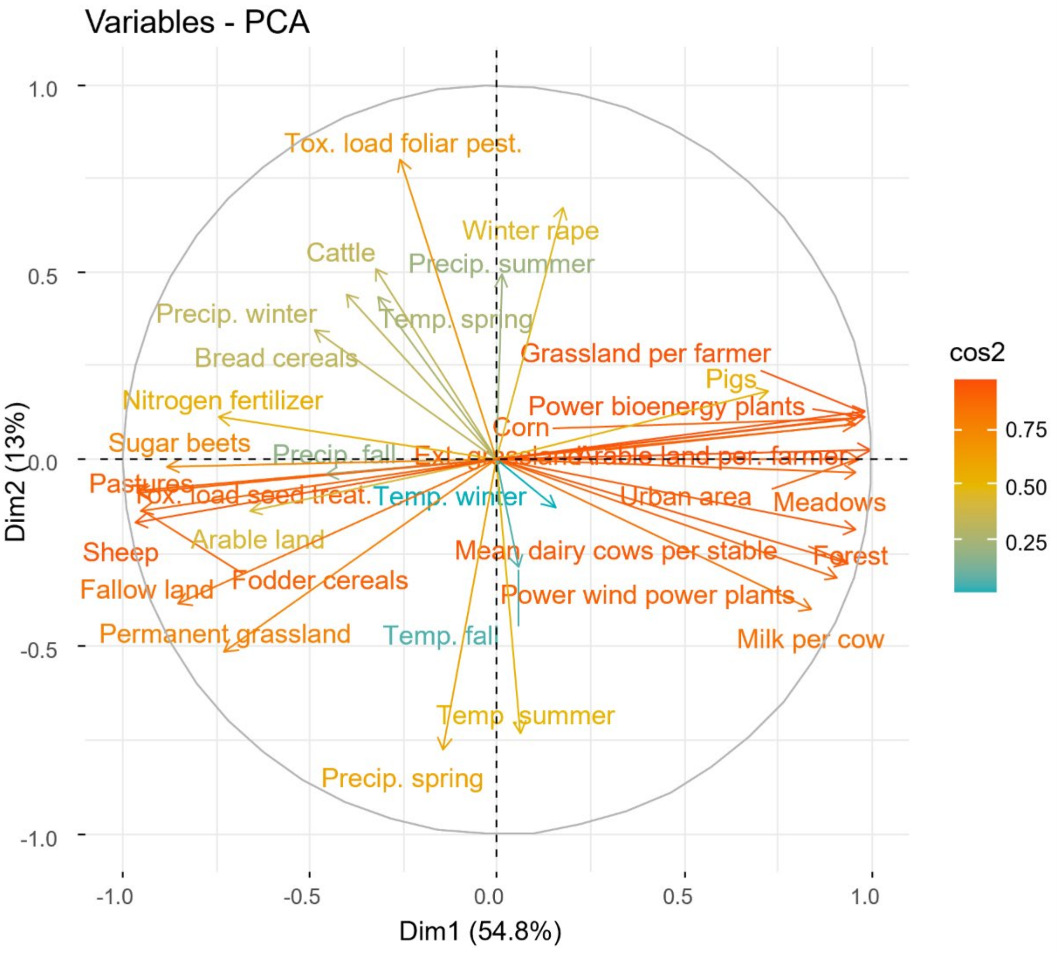

Figure S2. Biplot with visual representation of the relationships between variables for the full variable data set in the PCA. See Figure 1 for a detailed explanation of the plot components.

As in the reduced variable dataset, the PC2 was also heavily influenced by climate variables. In contrast to the reduced variable dataset, however, the main contributor of

the component was the newly added toxic load of pesticides in the agricultural landscape (Figure 3B). Additionally, a large amount of the variance in winter rape could be explained by PC2. Both, PC3 and PC4 were primarily influenced by single variables, namely amount of grown bread cereals and summer precipitation, respectively (Figure 3C,D).

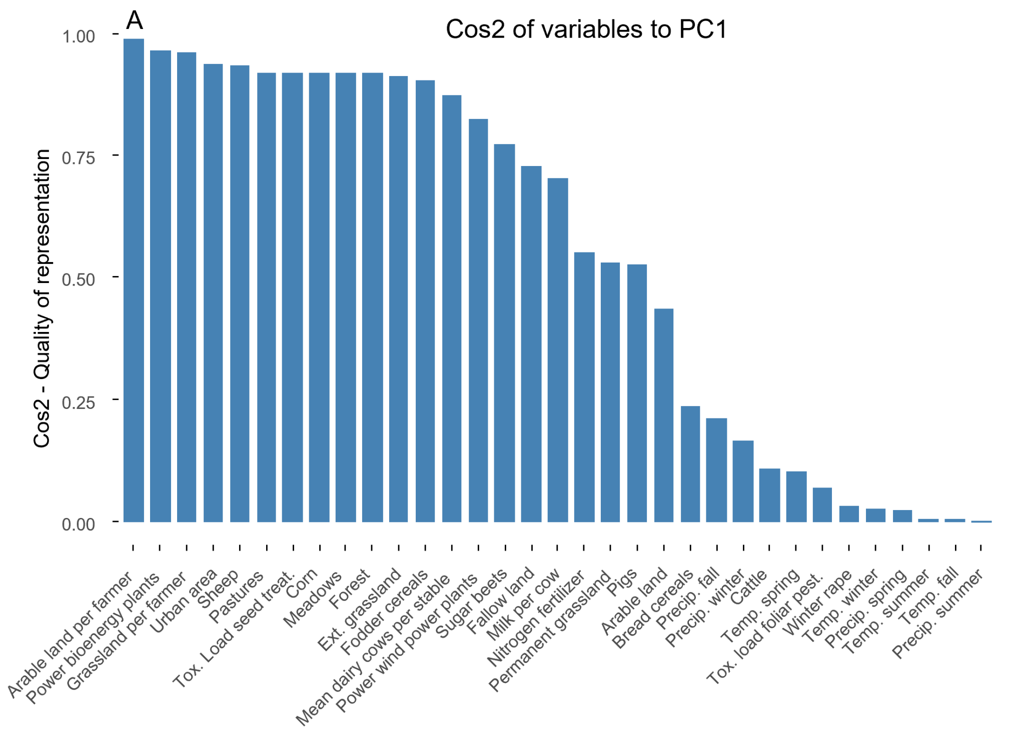
A
Cos2 of variables to PC1
Cos2 - Quality of representation
1.00
0.75
0.50
0.25
0.00
Arable land per farmer
Power bioenergy plants
Grassland per farmer
Urban area
Sheep
Pastures
Tox. Load seed treat.
Corn
Meadows
Forest
Ext. grassland
Fodder cereals
Mean dairy cows per stable
Power wind power plants
Sugar beets
Fallow land
Milk per cow
Nitrogen fertilizer
Permanent grassland
Pigs
Arable land
Bread cereals
Precip. fall
Precip. winter
Cattle
Temp. spring
Tox. load foliar pest.
Winter rape
Temp. winter
Precip. spring
Temp. summer
Temp. fall
Precip. summer

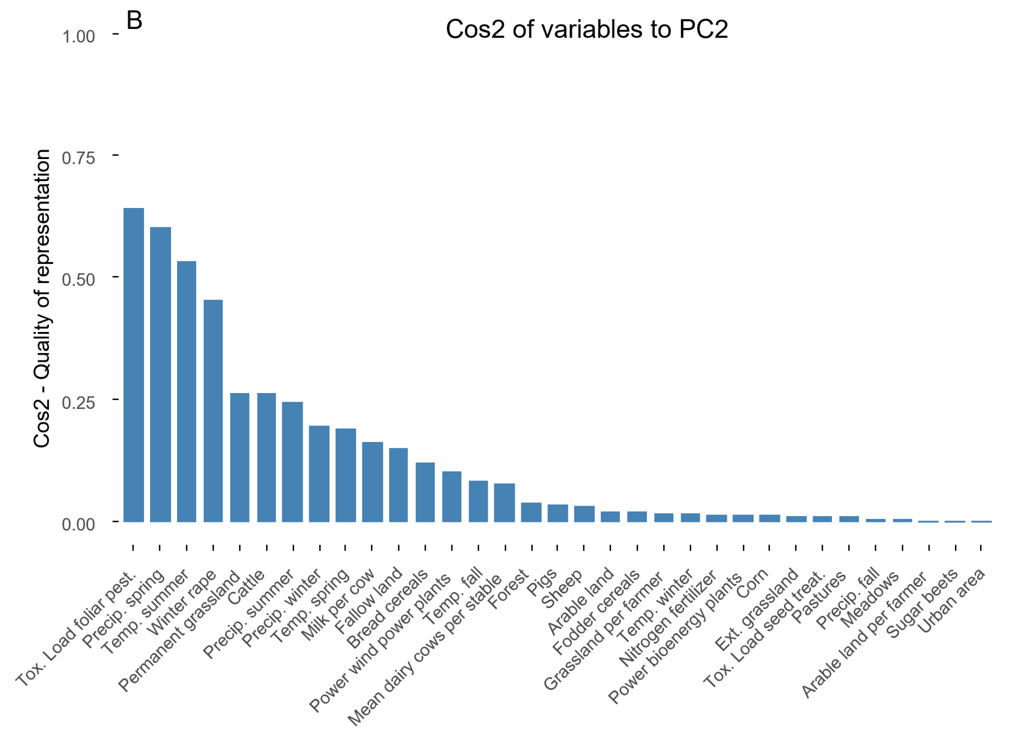
B
Cos2 of variables to PC2
Cos2 - Quality of representation
1.00
0.75
0.50
0.25
0.00
Tox. Load foliar pest.
Precip. spring
Temp. summer
Winter rape
Permanent grassland
Cattle
Precip. summer
Precip. winter
Temp. spring
Milk per cow
Fallow land
Bread cereals
Power wind power plants
Temp. fall
Mean dairy cows per stable
Forest
Pigs
Sheep
Arable land
Fodder cereals
Grassland per farmer
Temp. winter
Nitrogen fertilizer
Power bioenergy plants
Corn
Ext. grassland
Tox. Load seed treat.
Pastures
Precip. fall
Meadows
Arable land per farmer
Sugar beets
Urban area

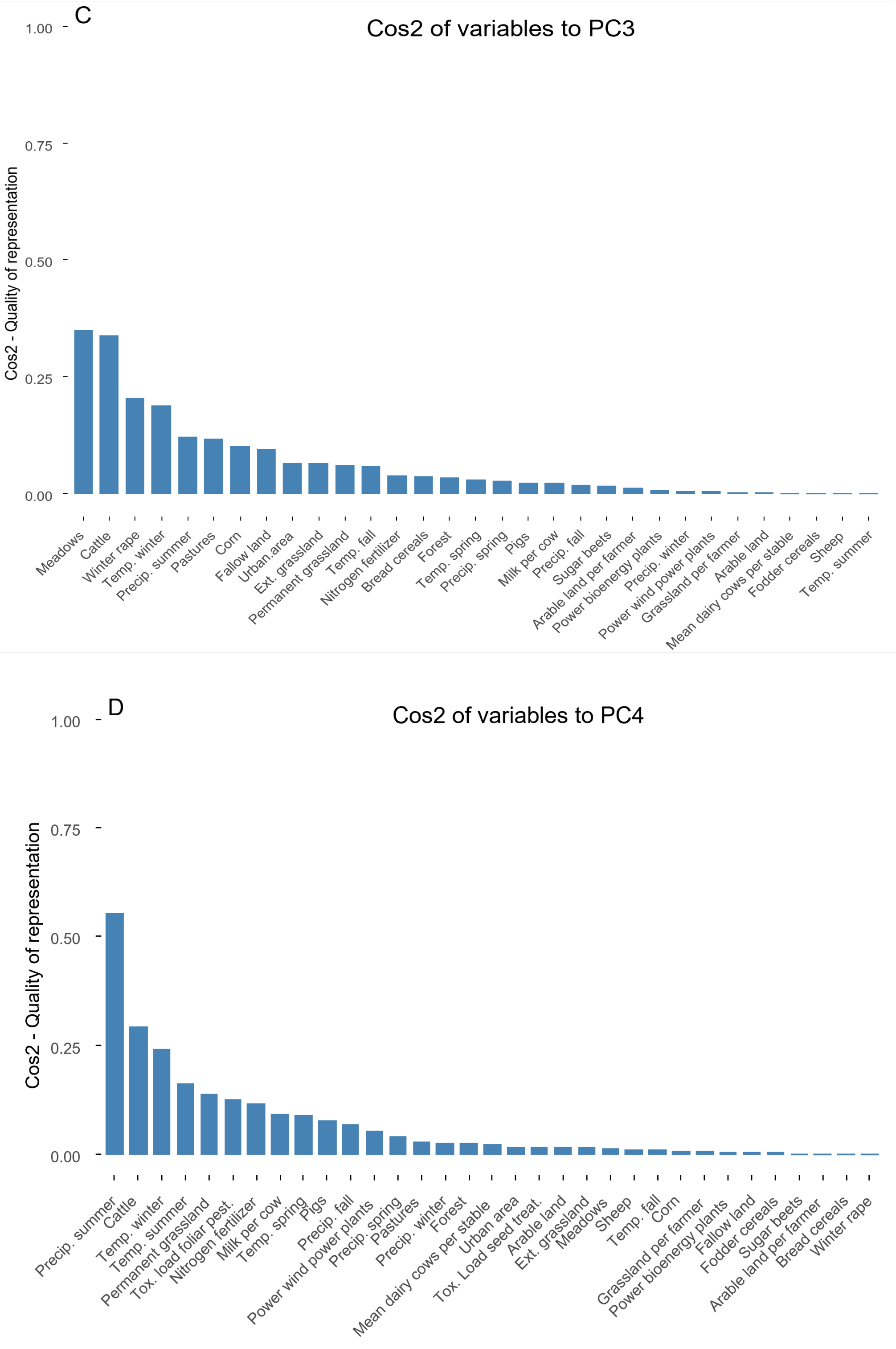

Figure S3 A-D depict cosine square values (cos2) for all variables in the full variable dataset for principal components 1 - 4.

The results of the linear model with the full variable dataset indicated that PC1 was a significant negative predictor of insect biomass (Table 1), suggesting that higher values of this agricultural intensification and land use change gradient are associated with lower insect biomass. Interestingly, higher toxic loads of seed treatments were also linked to higher FIB. In contrast, neither PC2, PC3, nor PC4 showed significant effects in the model, indicating that these components do not play a meaningful role in explaining variations in insect biomass within this analysis.

The overall model demonstrated a multiple R-squared value of 0.46, suggesting that the retained principal components explain a moderate portion of the variance in insect biomass.

Variable	Estimate	Std. Error	t	p
PC1	-0.23	0.08	-2.8	0.01 *
PC2	-0.17	0.16	-1.1	0.31
PC3	0.29	0.22	1.4	0.20
PC4	-0.04	0.23	-0.2	0.87

*Table S1. The estimated coefficients, standard errors, t-values, and p-values for the linear regression model. The model was fitted to the full variable dataset, with insect biomass as dependent, and principal components 1 to 4 as independent variables. Significant effects are indicated by * for p-values less than 0.05 and trends by . for p-values less than 0.1.*
